# Supplementary material for: Functional convergence of genomic and transcriptomic architecture underlies schooling behaviour in a live-bearing fish
Source: Nat Ecol Evol. 2023 Nov 20;8(1):98–110. doi: 10.1038/s41559-023-02249-9 (PMC10781616; doi:10.1038/s41559-023-02249-9)
Supplement: Supplementary file 1 — Supplementary Figs. 1–5, Tables 1–10 and references. [file 41559_2023_2249_MOESM1_ESM.pdf]

nature ecology & evolution

Article https://doi.org/10.1038/s41559-023-02249-9

Functional convergence of genomic and transcriptomic architecture underlies schooling behaviour in a live-bearing fish

In the format provided by the authors and unedited

Supplementary Figures

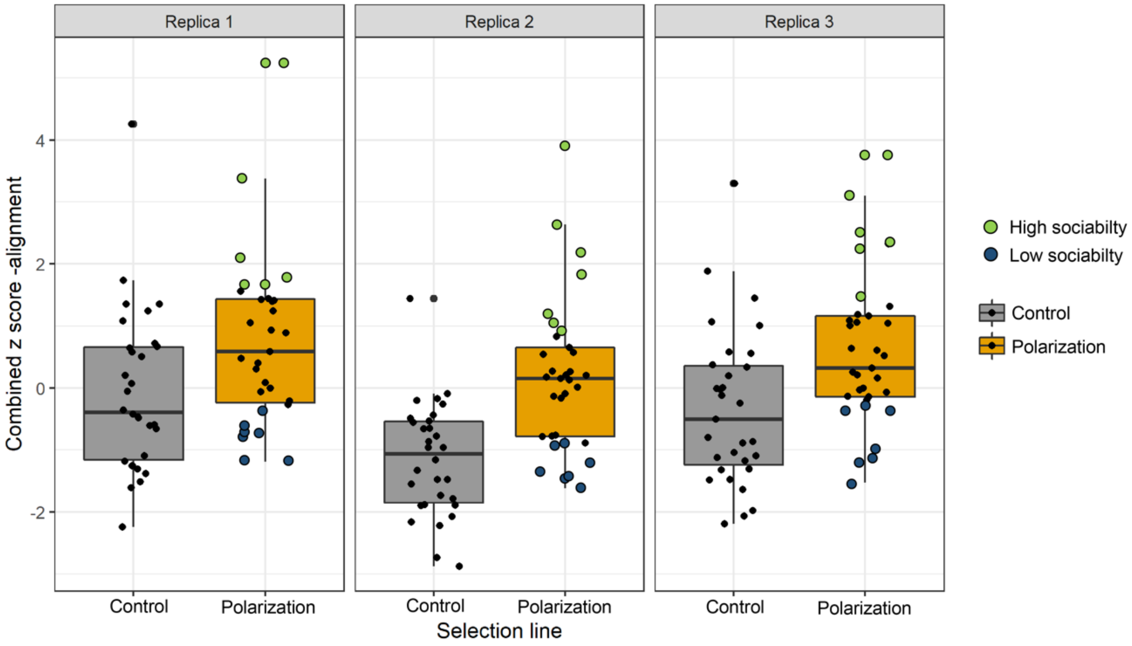

Supplementary Figure 1. Mean score for alignment to group direction in females for each of the 195 families measured when evaluating the heritability of sociability by means of open field tests in which guppies from three independently replicated polarization-selected (orange, n = 100) and control (gray, n = 95) lines were exposed to a group of other seven conspecifics. We obtained combined Z scores by standardizing independently alignment scores obtained in maternal and offspring generations and by adding the score of the mother to the mean score of all female offspring measure in each family. For all boxplots, horizontal lines indicate medians, boxes indicate the interquartile range, and whiskers indicate all points within 1.5 times the interquartile range. Colored circles indicate families that were further used for genomic analyses. Source data are provided as a Source Data file.

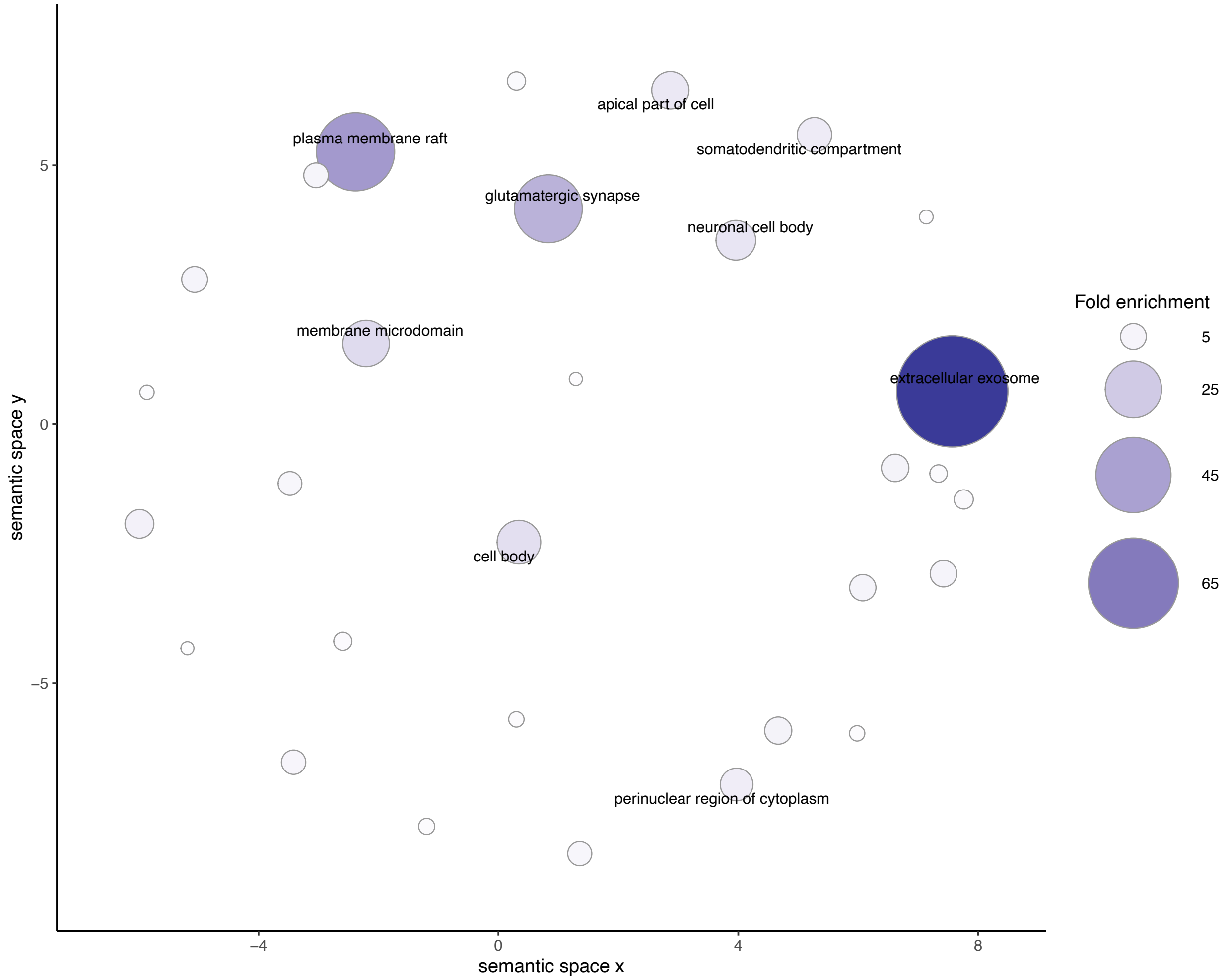

Supplementary Figure 2. Clustering of statistically significant overrepresented Gene Ontology annotations for cellular components associated to differences between high and low sociability in guppies. Point size and color provide information on fold enrichment value from the statistical overrepresentation test performed in PANTHER[1]. Terms with fold enrichment lower than eight are represented but not described in text. Axes have no intrinsic meaning and are based on multidimensional scaling which cluster terms based on semantic similarities[2]. Source data are provided as a Source Data file.

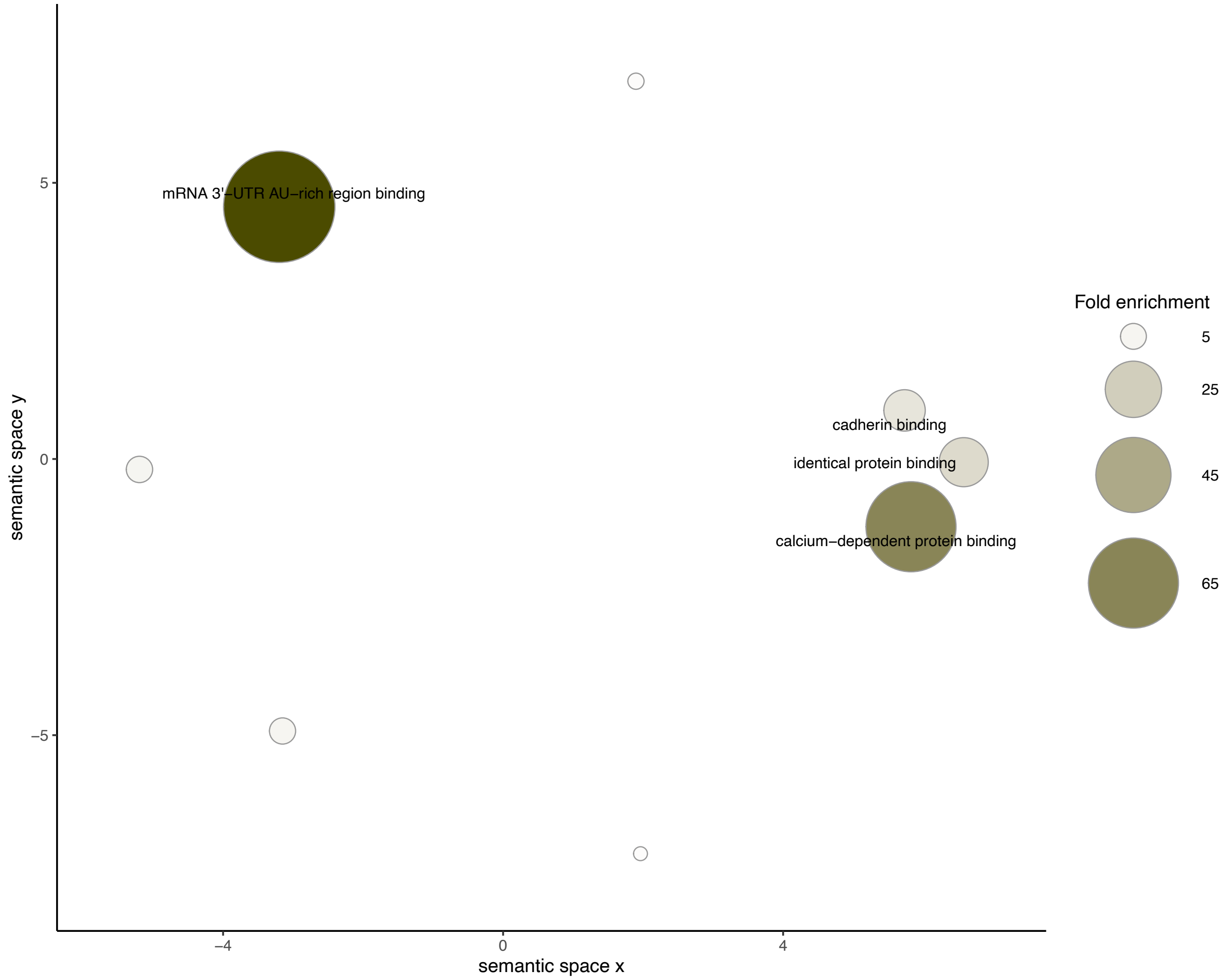

Supplementary Figure 3. Clustering of statistically significant overrepresented Gene Ontology annotations for molecular functions associated to differences between high and low sociability in guppies. Point size and color provide information on fold enrichment value from the statistical overrepresentation test performed in PANTHER[1]. Terms with fold enrichment lower than eight are represented but not described in text. Axes have no intrinsic meaning and are based on multidimensional scaling which cluster terms based on semantic similarities[2]. Source data are provided as a Source Data file.

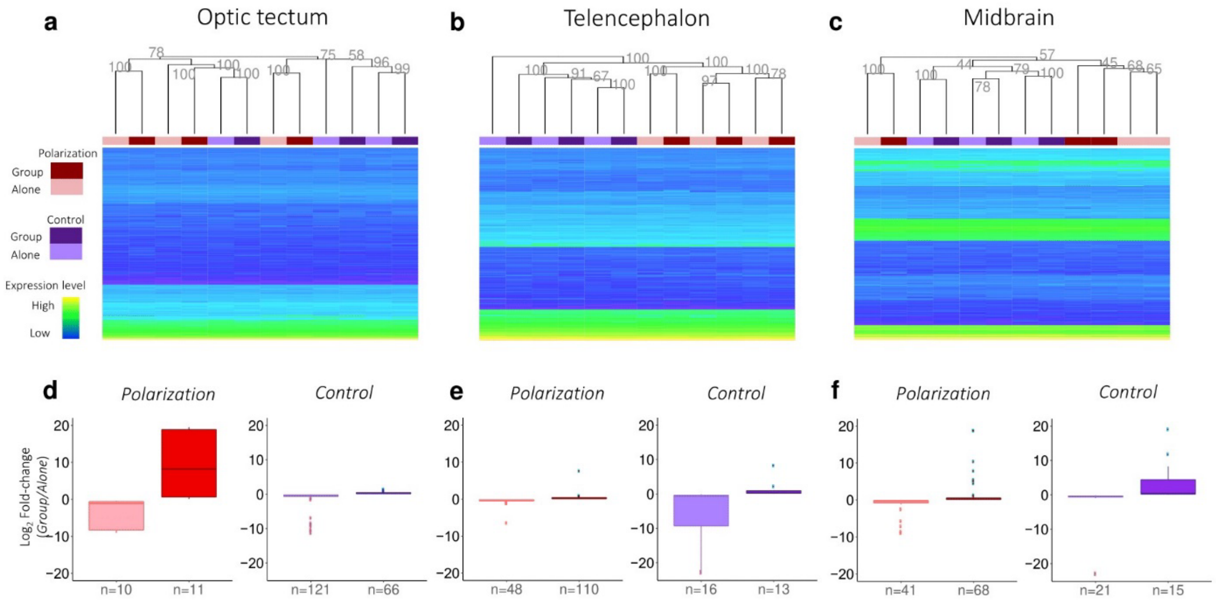

Supplementary Figure 4. Top panels: Hierarchical clustering of gene expression across all significantly expressed genes in the optic tectum (a), telencephalon (b) and midbrain (c) for polarization-selected selection and control lines. Values on top of nodes correspond to Approximately Unbiased bootstrap values. Heatmap represents expression of 500 randomly selected genes. **Bottom panels:** Boxplots of average log2 fold-change for all genes differentially expressed genes between the *Alone* and the *Group* conditions in polarization-selected and control lines for optic tectum (d), telencephalon (e) and midbrain (f). Sample size for each selection/treatment/tissue combination is provided in x-axis. Boxes correspond to 25th - 75th percentiles, whiskers indicate all points within 1.5 times the interquartile range. Sample sizes on the x-axis indicate number of genes with relative up- or down-regulation. Source data are provided as a Source Data file.

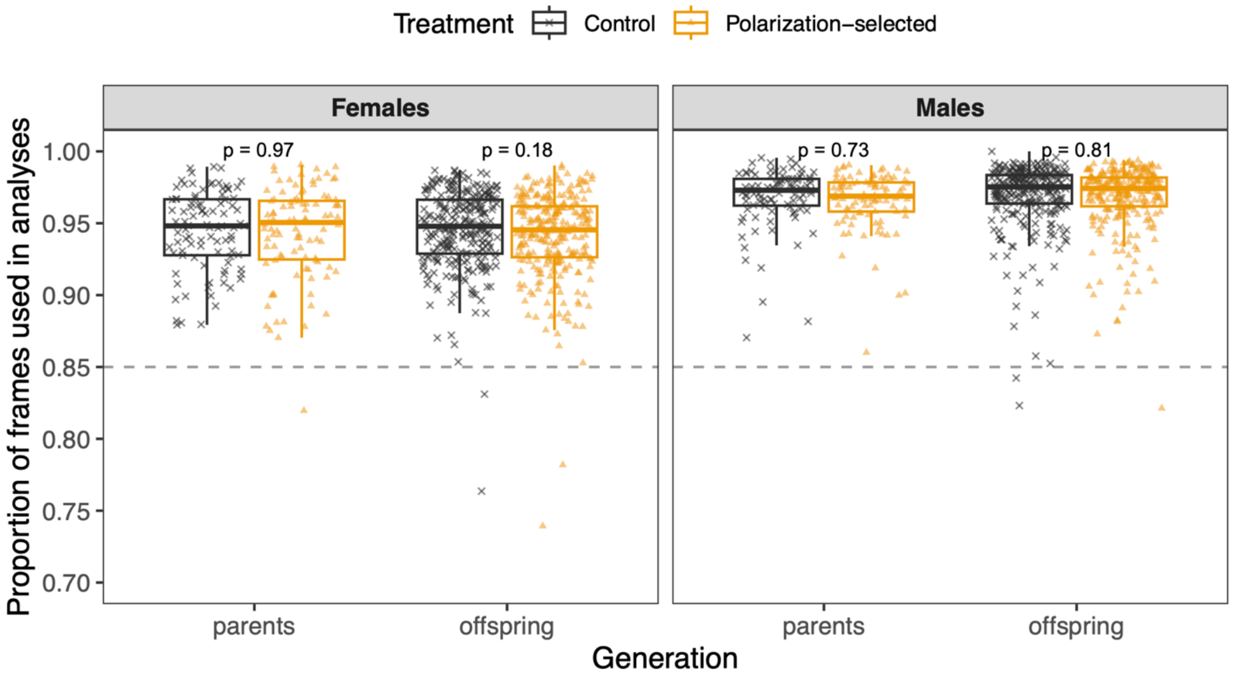

Supplementary Figure 5. Proportion of frames obtained from idTracker software in tracking from videos recorded for groups of seven wild-type guppies and one individual from either polarization-selected or control selection lines. Tracking data was used next for the quantification of alignment, attraction and speed in parents and six offspring of 195 families (Polarization-selected: n = 763; Control: n = 724). We found no significant differences for the proportion of frames used for videos used for polarization-selected and control lines in a Linear Mixed Model that included generation, sex and selection line as fixed factors (LMM_{frames}: line: F= 0.82; df = 1464; p = 0.37). Horizontal lines indicate medians, boxes indicate the interquartile range, and whiskers indicate all points within 1.5 times the interquartile range. P-values in top position of each comparison indicate values for statistical contrasts of the model by sex and generation. Consistency in identity assigned by idTracker was verified in trials with less than 85% frames tracked (dashed line). Source data are provided as a Source Data file.

Supplementary Tables

Supplementary Table 1. Statistical results for a Linear Mixed Model comparing the alignment and attraction of polarization-selected (n = 763) and control (n = 724) female guppies when swimming with groups of same-sex non-kin conspecifics. In bold p-values lower than 0.05 (see Supplementary code on shoaling patterns analyses).

	**Alignment to group**				
Predictors	*Estimates*	*CI*	*Statistic*	*p*	*df*
(Intercept)	0.19	0.13 – 0.24	6.78	**<0.001**	1385.81
Selection line [polarization]	0.01	0.00 – 0.01	2.27	**0.047**	9.68
Sex [males]	-0.01	-0.01 – -0.00	-3.02	**0.003**	690.08
Generation [parental]	-0.02	-0.02 – -0.01	-10.13	**<0.001**	1141.24
Average alignment of group	0.79	0.72 – 0.86	22.71	**<0.001**	1436.72
Average speed of group	0.00	0.00 – 0.00	0.20	0.842	1429.55
Selection line [polarization] * Sex [males]	0.00	-0.00 – 0.00	0.20	0.842	1429.55
ICC	0.12				
N_{line}	2				
N_{rep}	3				
N_{testnr}	8				
N_{sex}	2				
N_{family}	195				
Observations	1487				
Marginal R^2 / Conditional R^2	0.657 / 0.697				

	**Nearest neighbour distance (attraction)**				
Predictors	*Estimates*	*CI*	*Statistic*	*p*	*df*
(Intercept)	2.64	2.56 – 2.71	65.62	**<0.001**	565.12
Selection line [polarization]	-0.04	-0.08 – -0.00	-2.33	**0.044**	9.34

Sex [males]	0.01	-0.02 – 0.04	0.55	0.583	448.53
Generation [parental]	0.12	0.10 – 0.14	11.31	**<0.001**	984.50
Average alignment of group	0.02	0.02 – 0.02	27.31	**<0.001**	1408.27
Average speed of group	0.00	0.00 – 0.00	3.74	**<0.001**	566.82
Selection line [polarization] * Sex [males]	0.03	-0.02 – 0.07	1.24	0.217	371.59
ICC	0.13				
N_{line}	2				
N_{rep}	3				
N_{testnr}	8				
N_{sex}	2				
N_{family}	195				
Observations	1487				
Marginal R^2 / Conditional R^2	0.471 / 0.538				

Supplementary Table 2. Independent contrasts of a Linear Mixed Model evaluating potential differences between polarization-selected (P, n = 763) and control (C, n = 724) guppies in their alignment and nearest neighbor distance (attraction) when swimming with groups of same-sex non-kin conspecifics (see Supplementary code on shoaling patterns analyses).

Alignment

contrast	*sex*	*estimate*	*SE*	*df*	*t.ratio*	*p.value*
C - P	females	-0.006	0.002	9.68	-2.269	**0.047**
C - P	males	-0.003	0.002	9.56	1.378	0.20

Attraction

contrast	*treatment*	*estimate*	*SE*	*df*	*t.ratio*	*p.value*
C - P	females	0.042	0.001	9.34	2.331	**0.043**
C - P	males	0.015	0.002	9.26	0.883	0.40

Supplementary Table 3. Mean body size of guppies used to estimate the heritability of alignment and attraction at the time of testing across sexes and generations. Values are provided in number of pixels obtained from idTracker data using a custom script implemented in Matlab (v2020a) that extracts body size data accounting for changes in apparent size between the middle and edge of the experimental arena used for video recordings.

Generation	Selection line	Sex	Mean body size (pixels)	SE
Parents	Control	Females	410	47
		Males	192	29
	Polarization-selected	Females	416	71
		Males	189	37
Offspring	Control	Females	247	55
		Males	158	33
	Polarization-selected	Females	251	60
		Males	160	26

Supplementary Table 4. Results from Bayesian animal models only accounting for same-sex pedigree relationships. We obtained heritability estimates by taking the ratio of the additive genetic variance to the total phenotypic variance (additive genetic variance + group variance + population variance + residual variance) in each independent model.

ALIGNMENT

FEMALES

Parameter group	Parameter	Estimate	Est.Error	Q2.5	Q97.5
Fixed effects	Intercept	0.504	0.074	0.359	0.648
Fixed effects	linesocial	0.157	0.102	-0.04	0.355
Fixed effects	generationparents	-0.277	0.058	-0.39	-0.163
Fixed effects	speed_std	0.396	0.023	0.35	0.441
Additive genetic variance	Intercept	0.162	0.042	0.086	0.25
Group	Intercept	0.005	0.006	0	0.02
Population	Intercept	0.01	0.027	0	0.064
Residual variance	NA	0.304	0.037	0.234	0.379
**Heritability**		0.336	0.079	0.183	0.494

MALES

Parameter group	Parameter	Estimate	Est.Error	Q2.5	Q97.5
Fixed effects	Intercept	-0.452	0.125	-0.694	-0.213
Fixed effects	linesocial	0.093	0.172	-0.231	0.425
Fixed effects	generationparents	-0.502	0.071	-0.641	-0.362
Fixed effects	speed_std	0.298	0.039	0.221	0.376
Additive genetic variance	Intercept	0.031	0.026	0.000	0.091
Group	Intercept	0.04	0.016	0.013	0.074
Population	Intercept	0.039	0.091	0.001	0.207
Residual variance	NA	0.390	0.033	0.323	0.453
**Heritability**		0.063	0.052	0.000	0.184

ATTRACTION

FEMALES

Parameter group	Parameter	Estimate	Est.Error	Q2.5	Q97.5
Fixed effects	Intercept	-0.614	0.099	-0.811	-0.424
Fixed effects	linesocial	-0.088	0.138	-0.35	0.179
Fixed effects	generationparents	0.251	0.070	0.113	0.390
Fixed effects	speed_std	-0.109	0.024	-0.157	-0.062
Additive genetic variance	Intercept	0.094	0.038	0.025	0.174
Group	Intercept	0.038	0.015	0.011	0.071
Population	Intercept	0.023	0.061	0.000	0.133
Residual variance	NA	0.355	0.039	0.280	0.435

		Estimate	Est.Error	Q2.5	Q97.5
**Heritability**		0.185	0.072	0.049	0.336

MALES

Parameter group	Parameter	Estimate	Est.Error	Q2.5	Q97.5
Fixed effects	Intercept	0.476	0.134	0.212	0.746
Fixed effects	linesocial	-0.025	0.181	-0.389	0.345
Fixed effects	generationparents	0.614	0.088	0.440	0.785
Fixed effects	speed_std	0.054	0.046	-0.037	0.145
Additive genetic variance	Intercept	0.137	0.049	0.047	0.242
Group	Intercept	0.075	0.023	0.036	0.126
Population	Intercept	0.046	0.097	0.000	0.250
Residual variance	NA	0.454	0.050	0.359	0.556
**Heritability**		0.194	0.069	0.065	0.337

Supplementary Table 5 Results from Bayesian animal models with full pedigree relationships. We obtained heritability estimates for each sex by taking the ratio of the additive genetic variance of each sex to the total phenotypic variance (additive genetic variance + group variance + population variance + residual variance) in each independent model.

ALIGNMENT

Parameter group	Parameter	Estimate	Est.Error	Q2.5	Q97.5
Fixed effects	Intercept	0.52	0.102	0.331	0.718
Fixed effects	sexm	-0.961	0.063	-1.08	-0.838
Fixed effects	linesocial	0.163	0.128	-0.097	0.419
Fixed effects	speed_std	0.36	0.021	0.319	0.4
Fixed effects	sexm:linesocial	-0.075	0.074	-0.22	0.07
Fixed effects	sexf:generationparents	-0.31	0.066	-0.444	-0.181
Fixed effects	sexm:generationparents	-0.479	0.064	-0.604	-0.352
Additive genetic variance	sexf	0.105	0.029	0.051	0.166
Additive genetic variance	sexm	0.048	0.027	0.003	0.105
Additive genetic variance	cross (sexf x sexm)	0.583	0.270	0.021	0.974
Group	Intercept	0.025	0.009	0.009	0.045
Population	Intercept	0.023	0.051	0.001	0.13
Residual variance	NA	0.365	0.026	0.314	0.415
	♀ **Heritability**	0.202	0.052	0.102	0.308
	♂ **Heritability**	0.103	0.057	0.007	0.224
	**Δ Heritability** (♀ - ♂)	0.098	0.053	0.202	-0.005

ATTRACTION

Parameter group	Parameter	Estimate	Est.Error	Q2.5	Q97.5

Fixed effects	Intercept	-0.627	0.115	-0.847	-0.391
Fixed effects	sexm	1.09	0.075	0.942	1.23
Fixed effects	linesocial	-0.095	0.157	-0.425	0.219
Fixed effects	speed_std	-0.067	0.022	-0.109	-0.024
Fixed effects	sexm:linesocial	0.072	0.082	-0.091	0.235
Fixed effects	sexf:generationparents	0.283	0.079	0.129	0.438
Fixed effects	sexm:generationparents	0.568	0.080	0.411	0.722
Additive genetic variance	sexf	0.050	0.025	0.007	0.104
Additive genetic variance	sexm	0.176	0.041	0.102	0.263
Additive genetic variance	sexm	0.438	0.304	-0.168	0.950
Group	Intercept	0.059	0.013	0.035	0.087
Population	Intercept	0.032	0.056	0.002	0.172
Residual variance	NA	0.406	0.028	0.351	0.460
	♀ **Heritability**	0.092	0.044	0.013	0.188
	♂ **Heritability**	0.261	0.055	0.157	0.371
	**Δ Heritability (♀ - ♂)**	-0.169	0.051	-0.267	-0.069

Supplementary Table 6. Differentially expressed genes in GO term enrichment tests performed to evaluate the genomic response in midbrain, optic tectum and telencephalon in polarization-selected and control lines of female guppies when swimming with a group of conspecifics versus when swimming alone (two samples per treatment and brain region obtained by pooling brain tissue from 5 individuals each, n = 24 pools).

MIDBRAIN

CONTROL LINES (n=36)

Transcript	Gene BLAST	Average expression	Fold Change	P-value (FDR)
rna32553|gene=LOC103477857	PLCL2	131.212	27.132	<0.001
MSTRG.16482.1|gene=MSTRG.16482	MORN4	37.361	-22.976	<0.001
MSTRG.17365.1|gene=MSTRG.17365	RPGP2	32.181	-22.742	<0.001
rna23930|gene=ubap2l	ubap2l	944.324	-0.346	<0.001
rna14333|gene=trim33	trim33	41.183	19.099	<0.001
rna12705|gene=LOC103466075	MAGI2	1073.054	-0.398	<0.001
rna14220|gene=LOC103466954	FLNA	259.466	8.280	<0.001
rna43613|gene=LOC103458483	SYNE1	7853.703	0.243	<0.001
rna46407|gene=lrfn5	lrfn5	1152.716	-0.665	<0.001
rna12704|gene=LOC103466075	MAGI2	1284.870	0.358	0.001
MSTRG.18176.1|gene=MSTRG.18176	KMCP1	206.043	-0.800	0.001
rna32800|gene=u2af2	u2af2	1910.842	-0.346	0.001
MSTRG.16838.1|gene=MSTRG.16838	K1841	386.297	0.320	0.002
MSTRG.4096.1|gene=MSTRG.4096	AN13C	167.036	1.072	0.002
rna9959|gene=kcnab2	kcnab2	2321.886	-0.139	0.003
MSTRG.14391.4|gene=MSTRG.14391	KMT2A	2251.545	0.175	0.004
rna6890|gene=LOC103462605	IGF1R	151.381	5.464	0.004
MSTRG.16227.1|gene=MSTRG.16227	RIBA2	144.642	-0.623	0.004
rna27802|gene=fndc3a	fndc3a	639.741	-0.517	0.004
rna42558|gene=itpkb	itpkb	253.080	-0.714	0.006
MSTRG.6879.2|gene=MSTRG.6879	AEBP2	492.647	-0.492	0.009
rna15437|gene=LOC103467579	IQEC1	272.493	-0.544	0.010
MSTRG.10425.1|gene=MSTRG.10425	METK2	2522.539	-0.149	0.010
rna39037|gene=tanc2	tanc2	352.805	11.879	0.011
MSTRG.17415.2|gene=MSTRG.17415	FLOT1	1005.390	-0.284	0.018
MSTRG.3778.3|gene=MSTRG.3778	MAP1B	10822.870	0.090	0.022
rna19452|gene=LOC103470078	SCAM1	1692.812	0.214	0.027
rna30679|gene=LOC103476732	ANK3	6992.816	0.149	0.027
rna42097|gene=LOC103457647	NA	245.652	-0.955	0.027
MSTRG.7457.1|gene=MSTRG.7457	FLNA	1583.394	-0.378	0.033
MSTRG.7577.1|gene=MSTRG.7577	F19A5	1752.482	0.375	0.035
rna1276|gene=acsl1	acsl1	1052.049	-0.289	0.037

rna46838|gene=LOC103460240	FBL10	99.185	-0.667	0.038
rna18681|gene=LOC103469777	SMAD2	106.022	-0.567	0.042
rna14493|gene=wdr6	wdr6	151.669	0.864	0.048
rna1738|gene=keap1	keap1	2161.342	-0.225	0.049

POLARIZATION-SELECTED LINES (n=109)

Transcript	Gene BLAST	Average expression	Fold Change	P-value (FDR)
rna7061|gene=LOC103462879	PPR3E	144.810	10.467	< 0.001
rna9051|gene=LOC103463550	TLE4	90.600	-24.816	< 0.001
rna24376|gene=scn1b	scn1b	86.552	24.017	< 0.001
rna44020|gene=srrm1	srrm1	13.933	21.607	< 0.001
MSTRG.24305.1|gene=MSTRG.24305	NA	2510.567	0.748	< 0.001
rna22214|gene=egr1	egr1	30.831	18.839	< 0.001
rna22213|gene=egr1	egr1	30.831	18.839	< 0.001
rna40013|gene=LOC103482261	CPNE3	115.525	-1.047	< 0.001
rna45945|gene=syn3	syn3	2021.198	0.623	< 0.001
MSTRG.17634.6|gene=MSTRG.17634	CHIO	726.382	0.310	< 0.001
MSTRG.11465.4|gene=MSTRG.11465	LAMP2	1545.620	0.231	< 0.001
MSTRG.13269.1|gene=MSTRG.13269	FBCD1	2483.148	0.135	< 0.001
MSTRG.24306.3|gene=MSTRG.24306	SYN3	3100.717	1.061	< 0.001
rna26833|gene=LOC103474460	NCPR	105.368	5.135	0.001
MSTRG.3790.1|gene=MSTRG.3790	NA	6932.462	0.120	0.001
rna46319|gene=rps14	rps14	6939.074	-0.112	0.001
MSTRG.25027.1|gene=MSTRG.25027	NA	440.681	0.287	0.001
rna29486|gene=ubb	ubb	1424.005	-0.204	0.001
MSTRG.3031.1|gene=MSTRG.3031	NRK2	907.778	-0.234	0.001
rna35365|gene=LOC103479452	UN13A	866.972	-0.478	0.001
rna13371|gene=znf746	znf746	1623.600	0.312	0.001
MSTRG.10067.35|gene=MSTRG.10067	KCC2B	7708.760	0.138	0.002
MSTRG.5587.1|gene=MSTRG.5587	NA	387.393	0.325	0.002
rna24078|gene=LOC103472856	SHSA7	1873.094	0.380	0.002
rna4922|gene=LOC103461631	PTPRN	6258.089	0.342	0.002
rna30660|gene=rgs7	rgs7	2386.077	-0.155	0.002
rna46774|gene=abca2	abca2	985.996	0.437	0.002
rna44214|gene=stxbp6	stxbp6	1369.630	0.560	0.002
MSTRG.14206.1|gene=MSTRG.14206	SMBT1	81.425	-8.926	0.002
rna31627|gene=LOC103477235	BTBD3	5069.169	0.085	0.003
MSTRG.22324.1|gene=MSTRG.22324	INT7	201.560	0.525	0.003
rna15437|gene=LOC103467579	IQEC1	262.086	-0.619	0.003
rna30743|gene=LOC103476767	PTPRE	830.200	-0.300	0.003

MSTRG.23588.1|gene=MSTRG.23588	SC5A1	268.214	0.409	0.003
rna18718|gene=sema4c	sema4c	225.613	-0.778	0.003
rna18418|gene=LOC103469455	ZN208	964.520	0.791	0.004
MSTRG.24653.1|gene=MSTRG.24653	ZN665	647.821	-0.301	0.004
rna7261|gene=prc1	prc1	69.432	-0.673	0.004
rna33374|gene=itga8	itga8	177.778	-0.492	0.006
rna14054|gene=LOC103466873	CPNE9	1292.066	-0.186	0.006
rna25858|gene=add1	add1	301.934	-1.200	0.007
rna41611|gene=abracl	abracl	53.205	7.912	0.007
rna8442|gene=tbc1d23	tbc1d23	532.141	0.310	0.009
MSTRG.15056.1|gene=MSTRG.15056	ST32A	275.300	0.492	0.009
rna2302|gene=LOC103472525	NEUL1	298.298	-0.452	0.009
MSTRG.10567.1|gene=MSTRG.10567	EDIL3	901.674	0.304	0.009
rna20215|gene=klf9	klf9	230.772	0.515	0.009
MSTRG.24002.1|gene=MSTRG.24002	KCNA1	5020.815	0.142	0.009
rna43122|gene=npas3	npas3	579.387	0.533	0.010
MSTRG.11745.1|gene=MSTRG.11745	P33MX	3488.184	0.108	0.010
rna28848|gene=gng8	gng8	189.567	-0.795	0.011
MSTRG.14250.1|gene=MSTRG.14250	TMTC3	9253.455	0.190	0.011
rna32670|gene=LOC103477783	PNRC2	6535.494	0.077	0.011
rna37128|gene=LOC103480432	COR1B	96.683	-7.060	0.011
rna836|gene=LOC103465321	PLAK	165.686	-2.434	0.012
MSTRG.22229.1|gene=MSTRG.22229	NA	989.886	0.301	0.012
rna14269|gene=LOC103466973	MYT1	150.362	-8.518	0.012
rna26517|gene=LOC103474357	NA	680.176	0.312	0.013
rna14680|gene=tardbp	tardbp	786.594	-0.256	0.015
rna18417|gene=LOC103469455	ZN208	987.995	-0.992	0.016
rna12053|gene=rpl27a	rpl27a	5598.155	-0.121	0.016
rna3113|gene=fam171b	fam171b	676.632	-0.622	0.017
rna10216|gene=LOC103464678	S12A5	465.767	-0.326	0.017
MSTRG.24178.2|gene=MSTRG.24178	AEBP2	406.228	-0.382	0.018
rna41338|gene=clg20hxorf23	clg20hxorf 23	38.754	-5.680	0.018
rna25124|gene=rpl37	rpl37	3795.051	-0.136	0.018
rna21184|gene=dusp1	dusp1	601.109	0.245	0.019
rna22048|gene=LOC103471326	NA	5028.797	0.222	0.021
MSTRG.7705.1|gene=MSTRG.7705	TADBP	659.341	0.309	0.021
rna15823|gene=LOC103467371	LFG2	6610.378	0.128	0.021
rna42117|gene=foxo3	foxo3	4839.429	0.112	0.021
rna15369|gene=baz2a	baz2a	2468.169	0.271	0.023
rna16768|gene=wbp11	wbp11	637.220	0.247	0.023
rna36928|gene=dgkk	dgkk	330.445	-0.856	0.024

rna8044|gene=uchl5	uchl5	1901.123	-0.123	0.025
rna12667|gene=erc1	erc1	1227.615	0.223	0.026
MSTRG.20354.5|gene=MSTRG.20354	DLG5	269.626	0.856	0.029
rna2024|gene=LOC103470494	GRIA2	2812.527	0.166	0.030
rna10139|gene=pdhb	pdhb	2640.130	-0.139	0.030
MSTRG.8108.1|gene=MSTRG.8108	143BA	11583.530	0.122	0.030
MSTRG.11772.3|gene=MSTRG.11772	RNF6	375.432	0.328	0.030
rna35260|gene=wdr47	wdr47	2309.695	-0.136	0.030
MSTRG.1635.3|gene=MSTRG.1635	NLGNX	1082.247	0.316	0.030
MSTRG.6043.23|gene=MSTRG.6043	NFASC	4875.050	0.157	0.031
rna19015|gene=LOC103469839	SNF5	558.868	-0.187	0.031
rna30678|gene=LOC103476732	ANK3	748.739	0.483	0.031
rna26037|gene=LOC103473957	STXB1	55589.294	0.068	0.031
rna27530|gene=LOC103474859	H33	10943.188	-0.110	0.034
rna8930|gene=LOC103463612	RORB	1423.800	0.184	0.034
rna31916|gene=rps27a	rps27a	10460.725	-0.096	0.035
rna41322|gene=eif2s3	eif2s3	2821.758	-0.139	0.035
rna29177|gene=LOC103475891	AIFM1	41.012	5.526	0.035
MSTRG.25212.1|gene=MSTRG.25212	PLXA3	597.785	0.488	0.037
MSTRG.10784.1|gene=MSTRG.10784	RYR1	25.899	1.375	0.039
rna6858|gene=LOC103462628	CHST8	1822.516	0.123	0.039
rna19888|gene=LOC103470302	NA	327.381	-0.746	0.040
MSTRG.11901.1|gene=MSTRG.11901	NA	1157.565	0.231	0.044
rna28113|gene=actn4	actn4	1503.002	-0.194	0.044
MSTRG.24804.1|gene=MSTRG.24804	ABCA2	1009.735	-0.353	0.045
MSTRG.15875.1|gene=MSTRG.15875	ARHGC	511.656	0.253	0.045
rna1297|gene=fbxo41	fbxo41	2701.441	0.134	0.046
rna11314|gene=LOC103465330	AGAP2	872.129	0.343	0.047
rna25393|gene=cdc37l1	cdc37l1	253.320	-0.270	0.047
rna43115|gene=akap6	akap6	659.763	4.534	0.047
MSTRG.9250.1|gene=MSTRG.9250	MFS6L	901.045	0.193	0.048
rna22955|gene=gatad2b	gatad2b	5809.079	0.156	0.048
MSTRG.1327.2|gene=MSTRG.1327	G137C	1064.576	0.197	0.048
MSTRG.17605.2|gene=MSTRG.17605	CEP72	4481.235	0.158	0.048
MSTRG.9514.1|gene=MSTRG.9514	KPCA	2230.300	0.158	0.049

OPTIC TECTUM

CONTROL LINES (n=187)

Transcript	Gene BLAST	Average expression	Fold Change	P-value (FDR)
rna31156|gene=LOC103476975	ERLN1	44.054	-23.947	< 0.001
rna45754|gene=kcnc2	kcnc2	862.523	0.336	< 0.001

MSTRG.3467.33|gene=MSTRG.3467	MADD	535.023	-0.426	< 0.001
MSTRG.15801.1|gene=MSTRG.15801	FEZ1	709.036	0.450	< 0.001
rna47175|gene=fsd1l	fsd1l	57.585	-6.841	< 0.001
rna8739|gene=LOC103463733	PAIRB	184.831	-8.877	0.001
rna11571|gene=nfasc	nfasc	204.340	-10.374	0.001
rna21659|gene=gsr	gsr	152.518	-0.791	0.001
MSTRG.14289.2|gene=MSTRG.14289	CLPB	860.114	-0.369	0.004
MSTRG.18014.5|gene=MSTRG.18014	SAC1A	583.077	-0.301	0.004
rna38083|gene=eme2	eme2	298.137	-0.519	0.004
MSTRG.5196.2|gene=MSTRG.5196	HEMK1	387.510	-1.001	0.004
rna28725|gene=proser1	proser1	414.492	-0.419	0.004
MSTRG.15730.1|gene=MSTRG.15730	MAP6	1424.326	-0.361	0.004
rna28934|gene=LOC103475715	NPAS2	236.645	-1.310	0.004
MSTRG.917.1|gene=MSTRG.917	MYG	2925.534	0.261	0.004
MSTRG.9656.1|gene=MSTRG.9656	MFS11	246.930	-0.343	0.005
rna12640|gene=sephs1	sephs1	61.114	-8.344	0.005
rna9975|gene=timp4	timp4	4018.568	0.133	0.005
rna21336|gene=sparc	sparc	2184.843	0.289	0.005
rna18695|gene=LOC103469766	XNIF	6926.725	-0.137	0.005
MSTRG.13682.4|gene=MSTRG.13682	DYN1	902.700	-0.436	0.005
rna39368|gene=LOC103481895	PHIPL	1250.523	0.275	0.006
MSTRG.24446.1|gene=MSTRG.24446	NA	129.841	-0.756	0.006
rna20093|gene=LOC103470431	rsmG	56982.959	0.171	0.006
MSTRG.10409.5|gene=MSTRG.10409	HIP1R	229.374	-1.111	0.007
MSTRG.24774.24|gene=MSTRG.24774	E41L3	222.455	-0.670	0.008
rna17061|gene=cacna1i	cacna1i	967.348	-0.420	0.009
rna19619|gene=pde4d	pde4d	389.720	-0.416	0.009
rna23861|gene=clk2	clk2	1467.038	-0.164	0.009
rna20745|gene=LOC103470831	S12A2	1144.392	0.736	0.009
MSTRG.22864.5|gene=MSTRG.22864	MABP1	646.420	-0.385	0.009
rna39712|gene=cacna1g	cacna1g	915.637	-0.347	0.010
MSTRG.10346.1|gene=MSTRG.10346	PERF	664.481	0.237	0.010
MSTRG.24190.1|gene=MSTRG.24190	NYNRI	734.469	-0.722	0.010
MSTRG.13834.2|gene=MSTRG.13834	NMDZ1	4878.659	-0.246	0.010
rna46689|gene=LOC103460147	E41L3	326.656	-0.451	0.010
rna1478|gene=suclg1	suclg1	310.395	0.424	0.010
rna18243|gene=LOC103469344	RHG23	663.109	0.551	0.010
MSTRG.16227.1|gene=MSTRG.16227	RIBA2	133.475	-0.668	0.010
rna11521|gene=LOC103465433	NA	3055.567	-0.322	0.010
rna32056|gene=kiaa0319	kiaa0319	425.023	-0.831	0.011
rna33697|gene=LOC103478428	FEZ2	157.395	-0.446	0.011

rna39112|gene=LOC103481688	ANK3	1151.108	-0.739	0.011
MSTRG.7552.1|gene=MSTRG.7552	GBRD	924.733	-0.409	0.011
rna6818|gene=chd9	chd9	506.594	1.474	0.012
MSTRG.6879.2|gene=MSTRG.6879	AEBP2	755.567	-0.594	0.013
rna8820|gene=bsg	bsg	5022.883	0.117	0.013
rna32896|gene=rbp5	rbp5	700.965	0.314	0.013
rna9941|gene=LOC103464464	NA	6700.276	0.142	0.013
rna4264|gene=s100b	s100b	44245.997	0.203	0.013
rna10292|gene=LOC103464714	SYT2	951.110	-0.683	0.013
rna32087|gene=LOC103477466	PI4KB	1081.824	-0.409	0.013
rna24933|gene=LOC103473353	SMCA2	1265.118	-0.322	0.013
rna8437|gene=gtpbp6	gtpbp6	407.444	-0.307	0.013
MSTRG.20719.13|gene=MSTRG.20719	SHSA6	1502.578	-0.408	0.013
rna43889|gene=LOC103458562	CO052	769.649	-0.511	0.013
rna31795|gene=rims1	rims1	871.867	-0.267	0.013
rna39949|gene=arhgef4	arhgef4	391.940	-0.310	0.013
MSTRG.14169.1|gene=MSTRG.14169	APBP2	489.681	-0.306	0.013
MSTRG.17087.27|gene=MSTRG.17087	SYGP1	2095.222	-0.290	0.013
MSTRG.18474.3|gene=MSTRG.18474	GDE	1577.365	-0.197	0.014
rna24455|gene=ago4	ago4	805.347	0.399	0.014
MSTRG.15686.3|gene=MSTRG.15686	SRRM3	873.289	-0.377	0.014
rna24998|gene=fnbp1	fnbp1	733.773	-0.604	0.015
rna27624|gene=serpine2	serpine2	3522.939	0.124	0.015
rna35717|gene=LOC103479568	PSA7	1970.107	0.113	0.015
rna5025|gene=LOC103460921	PDXK	3641.298	0.200	0.016
MSTRG.24191.1|gene=MSTRG.24191	EFC1	758.821	-0.790	0.016
MSTRG.19248.1|gene=MSTRG.19248	NALP1	250.455	-0.743	0.016
rna14471|gene=LOC103467107	VWA1	291.946	0.459	0.016
rna27096|gene=sik2	sik2	1497.974	-0.340	0.016
rna4809|gene=dapl1	dapl1	529.088	0.361	0.016
rna20030|gene=LOC103470390	LCAP	1033.226	0.321	0.017
rna385|gene=LOC103474313	HEBP2	6881.290	0.112	0.018
rna14585|gene=LOC103467181	GSTM1	383.607	0.332	0.018
MSTRG.1958.1|gene=MSTRG.1958	LRRF2	1052.046	-0.222	0.021
rna16860|gene=pick1	pick1	224.937	-0.839	0.021
MSTRG.9169.3|gene=MSTRG.9169	TTYH3	243.157	0.339	0.021
MSTRG.3724.24|gene=MSTRG.3724	SHAN2	960.854	-0.308	0.022
rna29486|gene=ubb	ubb	1938.270	0.196	0.023
rna35679|gene=LOC103479583	DPP6	226.201	-0.421	0.024
rna1353|gene=pgm2	pgm2	298.878	1.051	0.024
rna4718|gene=ndufs1	ndufs1	704.549	0.317	0.024

rna25185|gene=LOC103473483	SCRB2	1298.625	0.227	0.024
MSTRG.11218.1|gene=MSTRG.11218	CC142	510.828	-0.225	0.024
rna6428|gene=LOC103462451	TRI66	691.559	0.489	0.024
rna39783|gene=atad1	atad1	224.854	-0.367	0.024
rna39263|gene=LOC103481783	ZN235	294.711	-0.637	0.025
rna31608|gene=LOC103477221	CYT	2957.013	0.211	0.027
rna8962|gene=LOC103463594	ZFYV9	2108.108	-0.195	0.027
rna30529|gene=LOC103476656	GDS1	1925.695	0.332	0.027
MSTRG.4676.4|gene=MSTRG.4676	DOT1L	1214.736	-0.288	0.027
rna25497|gene=LOC103473681	NA	2400.413	0.321	0.027
rna33379|gene=LOC103478617	HEY1	707.379	0.232	0.028
rna42980|gene=gja1	gja1	24024.747	0.156	0.029
rna1352|gene=pgm2	pgm2	430.082	-0.541	0.029
MSTRG.4713.3|gene=MSTRG.4713	ZFYV9	3966.457	-0.290	0.029
rna5711|gene=mrpl46	mrpl46	342.311	0.276	0.029
MSTRG.6387.2|gene=MSTRG.6387	AASS	519.728	-0.348	0.029
rna9652|gene=ccdc51	ccdc51	89.289	0.739	0.029
MSTRG.4313.1|gene=MSTRG.4313	LORF2	40.998	1.225	0.029
MSTRG.14276.7|gene=MSTRG.14276	HIP1	502.571	-0.272	0.029
MSTRG.6781.1|gene=MSTRG.6781	NA	176.637	-0.375	0.029
rna34295|gene=aldh2	aldh2	948.974	0.186	0.029
MSTRG.8952.3|gene=MSTRG.8952	CAC1I	2257.156	-0.189	0.030
rna16138|gene=LOC103468055	L1CAM	2054.282	-0.230	0.030
rna17402|gene=rhbdf1	rhbdf1	461.449	-0.505	0.030
rna45|gene=fbxl18	fbxl18	1526.888	-0.201	0.030
MSTRG.21801.2|gene=MSTRG.21801	NA	161.643	-11.290	0.030
MSTRG.11045.2|gene=MSTRG.11045	STAG2	696.471	-0.327	0.030
MSTRG.18008.6|gene=MSTRG.18008	RBM42	981.920	-0.292	0.030
MSTRG.5146.19|gene=MSTRG.5146	CAPS1	2211.292	-0.368	0.031
rna554|gene=LOC103464924	NA	1135.002	0.275	0.031
MSTRG.22927.5|gene=MSTRG.22927	PANK2	199.419	-0.358	0.031
MSTRG.4337.1|gene=MSTRG.4337	NA	163.807	-0.398	0.031
MSTRG.6421.1|gene=MSTRG.6421	DNM1L	284.253	-0.331	0.032
MSTRG.20883.51|gene=MSTRG.20883	CAC1G	2176.919	-0.280	0.032
rna17397|gene=hbz	hbz	2654.700	0.777	0.032
rna9952|gene=pax7	pax7	2715.333	0.252	0.033
MSTRG.12285.7|gene=MSTRG.12285	CSPG5	498.929	-0.448	0.033
MSTRG.4508.3|gene=MSTRG.4508	ANR29	148.773	-0.558	0.034
rna15287|gene=LOC103467653	MYPT2	161.547	-0.529	0.034
MSTRG.21917.2|gene=MSTRG.21917	AIG1	812.919	-0.226	0.034
rna31978|gene=cul9	cul9	815.724	-0.454	0.034

MSTRG.5079.1|gene=MSTRG.5079	CCD66	158.970	-0.655	0.034
rna33477|gene=hdac9	hdac9	1208.808	-0.204	0.034
rna32208|gene=LOC103477566	NA	136.825	-8.874	0.034
MSTRG.23173.1|gene=MSTRG.23173	M3K9	393.992	-0.443	0.034
rna44660|gene=rcan3	rcan3	1297.437	0.176	0.034
MSTRG.22663.6|gene=MSTRG.22663	CNIH3	1399.089	-0.365	0.035
rna2408|gene=LOC103472140	STRN	534.708	-0.374	0.035
MSTRG.12275.1|gene=MSTRG.12275	NA	289.805	-0.465	0.035
MSTRG.11276.1|gene=MSTRG.11276	NA	808.305	0.259	0.035
MSTRG.6086.4|gene=MSTRG.6086	SETD5	646.909	-0.482	0.035
rna20492|gene=ncs1	ncs1	1548.351	0.135	0.035
rna44860|gene=mtpn	mtpn	3939.993	0.096	0.035
rna9563|gene=LOC103464301	SC6A6	3463.148	0.093	0.035
MSTRG.20597.113|gene=MSTRG.20597	ANK3	3978.012	-0.257	0.035
rna15537|gene=LOC103467937	ST1S3	92.866	0.506	0.035
MSTRG.2919.2|gene=MSTRG.2919	KPBB	1982.178	-0.275	0.035
MSTRG.8196.8|gene=MSTRG.8196	CDK17	523.422	-0.384	0.035
MSTRG.10756.1|gene=MSTRG.10756	CACP	73.826	1.141	0.036
MSTRG.21665.4|gene=MSTRG.21665	ASAP1	2262.217	-0.338	0.036
rna9074|gene=LOC103463535	SAFB2	486.492	0.877	0.036
MSTRG.6893.1|gene=MSTRG.6893	KCND2	751.620	-0.249	0.037
rna37092|gene=gabarap	gabarap	9554.826	0.122	0.037
MSTRG.21609.4|gene=MSTRG.21609	MYPT1	538.478	-1.474	0.037
MSTRG.17335.7|gene=MSTRG.17335	KPCA	1293.373	-0.233	0.037
MSTRG.14062.6|gene=MSTRG.14062	NCAM1	671.251	-0.400	0.038
rna48393|gene=LOC103461400	PPAL	460.373	0.240	0.039
rna43205|gene=ckb	ckb	568.475	0.225	0.039
rna46432|gene=ddx46	ddx46	2735.921	-0.244	0.039
rna10428|gene=LOC103464779	HES5	738.767	0.267	0.039
rna13605|gene=LOC103466450	RFX7	1889.887	0.384	0.039
rna1698|gene=LOC103468483	ELOF1	256.797	0.280	0.039
rna37063|gene=bcl6b	bcl6b	201.983	0.501	0.039
rna36246|gene=smg7	smg7	2826.022	-0.149	0.039
MSTRG.8764.2|gene=MSTRG.8764	DDX5	9210.083	-0.235	0.039
rna20266|gene=LOC103470511	DNM1L	1990.942	-0.169	0.039
MSTRG.8986.28|gene=MSTRG.8986	CAC1A	5587.949	-0.207	0.039
MSTRG.12290.2|gene=MSTRG.12290	CTNB1	2910.930	-0.206	0.039
rna564|gene=gde1	gde1	785.583	0.260	0.041
MSTRG.16875.3|gene=MSTRG.16875	S22AF	106.886	-0.823	0.041
MSTRG.11867.7|gene=MSTRG.11867	GRIA2	7579.132	-0.254	0.041
rna28723|gene=thsd1	thsd1	124.740	-0.707	0.042

rna32852|gene=casp2	casp2	194.575	0.487	0.042
MSTRG.19932.2|gene=MSTRG.19932	ERF3A	147.054	-0.532	0.042
MSTRG.14143.1|gene=MSTRG.14143	WSB1	2604.092	-0.329	0.043
MSTRG.1695.1|gene=MSTRG.1695	WBP4	118.747	-0.790	0.043
rna12783|gene=cib1	cib1	172.780	0.314	0.045
rna39046|gene=LOC103481669	RHG27	219.443	0.377	0.045
rna4111|gene=etv5	etv5	177.296	0.799	0.045
rna45179|gene=LOC103459268	CAC1C	1903.168	-0.854	0.046
rna43863|gene=zbtb42	zbtb42	95.269	-10.618	0.047
rna43182|gene=tyro3	tyro3	1426.998	0.392	0.047
rna26654|gene=snx19	snx19	2368.539	-0.171	0.047
rna16854|gene=LOC103468448	BORG5	259.020	0.308	0.048
MSTRG.8096.18|gene=MSTRG.8096	WNK2	3178.569	-0.321	0.049
MSTRG.17167.1|gene=MSTRG.17167	ARL4A	107.263	-9.441	0.049
MSTRG.10976.1|gene=MSTRG.10976	NA	339.637	-0.260	0.049
rna18354|gene=LOC103469494	EIF1	480.176	-0.425	0.049
rna12804|gene=slc1a2	slc1a2	38882.612	0.102	0.049
MSTRG.5171.1|gene=MSTRG.5171	RBM5	1463.233	-0.303	0.049
MSTRG.18686.1|gene=MSTRG.18686	RHG12	753.715	-0.292	0.049
MSTRG.24504.1|gene=MSTRG.24504	RAE1	743.520	-0.294	0.049
MSTRG.3297.5|gene=MSTRG.3297	DGLA	461.380	-0.328	0.049

POLARIZATION-SELECTED LINES (n=21)

Transcript	Gene BLAST	Average expression	Fold Change	P-value (FDR)
MSTRG.21801.2|gene=MSTRG.21801	NA	102.640	24.315	< 0.001
rna20241|gene=entpd4	entpd4	13.232	21.501	< 0.001
rna5604|gene=cdh8	cdh8	1553.154	0.643	< 0.001
rna24376|gene=scn1b	scn1b	92.098	19.494	< 0.001
rna22214|gene=egr1	egr1	39.104	18.875	< 0.001
rna22213|gene=egr1	egr1	39.104	18.875	< 0.001
MSTRG.2915.2|gene=MSTRG.2915	CADH8	292.560	-7.961	< 0.001
rna15769|gene=LOC103467406	DSLE	156.024	-0.735	0.002
MSTRG.22698.1|gene=MSTRG.22698	LAMA4	269.181	-8.539	0.004
rna29823|gene=wwc1	wwc1	283.825	-9.054	0.008
rna1269|gene=gucy1a3	gucy1a3	206.417	-0.773	0.010
rna28422|gene=LOC103475388	CTR3	1020.217	0.605	0.015
rna35022|gene=LOC103479254	KC1G2	174.252	-1.351	0.020
rna16375|gene=LOC103468166	MPP2	287.359	0.758	0.020
rna29120|gene=LOC103475822	SPTB2	245.255	-8.404	0.032
rna12010|gene=slc6a15	slc6a15	4110.120	0.134	0.034

rna23231|gene=ahdc1	ahdc1	875.602	-0.421	0.034
rna35400|gene=LOC103479433	P66B	129.015	-0.435	0.034
rna39880|gene=tmem94	tmem94	138.800	8.215	0.040
rna7779|gene=LOC103463266	RAB3C	271.999	-0.557	0.040
rna41072|gene=LOC103456849	ESCO1	357.308	11.958	0.040

TELENCEPHALON

CONTROL LINES (n=29)

Transcript	Gene BLAST	Average expression	Fold Change	P-value (FDR)
rna16008|gene=zmynd12	zmynd12	131.990	3< 0.001	< 0.001
rna45464|gene=slc37a3	slc37a3	38.721	-22.872	< 0.001
rna14004|gene=LOC103466852	CNGB1	40.820	22.427	< 0.001
rna36260|gene=LOC103480019	PTPR2	26.631	-22.537	< 0.001
MSTRG.16482.1|gene=MSTRG.16482	MORN4	15.553	-22.396	< 0.001
rna9271|gene=ncln	ncln	35.830	8.337	< 0.001
rna1296|gene=fbxo41	fbxo41	364.240	-0.593	< 0.001
rna10792|gene=plxna1	plxna1	299.891	1.086	0.001
rna34474|gene=LOC103478864	HHATL	249.894	0.719	0.003
MSTRG.16496.1|gene=MSTRG.16496	LDB1	437.207	-0.439	0.006
rna8510|gene=cc2d2a	cc2d2a	231.497	-0.488	0.007
rna45291|gene=grip1	grip1	1272.284	0.304	0.009
rna564|gene=gde1	gde1	945.463	0.225	0.009
rna752|gene=tsc2	tsc2	572.522	-0.466	0.014
rna33839|gene=LOC103478362	COBL	316.960	0.373	0.015
rna38088|gene=LOC103481105	VATL	241.247	-0.797	0.015
MSTRG.10409.5|gene=MSTRG.10409	HIP1R	136.542	-1.063	0.016
MSTRG.20355.9|gene=MSTRG.20355	KCMA1	1362.688	-0.259	0.017
MSTRG.21801.2|gene=MSTRG.21801	NA	148.516	-8.653	0.018
rna13396|gene=islr2	islr2	11284.671	0.081	0.020
MSTRG.22908.2|gene=MSTRG.22908	ATRN	287.114	-0.510	0.020
MSTRG.22993.1|gene=MSTRG.22993	SSBP3	1070.362	0.254	0.020
rna2770|gene=map4k4	map4k4	1829.434	0.204	0.020
MSTRG.205.3|gene=MSTRG.205	IFT27	76.967	2.301	0.021
rna45311|gene=ptprb	ptprb	221.601	-10.953	0.027
rna28655|gene=LOC103475549	TRIM3	234.203	0.738	0.027
rna26416|gene=LOC103474211	ROBO2	1300.445	-0.301	0.044
MSTRG.23960.3|gene=MSTRG.23960	RB6I2	643.993	-0.251	0.046
rna16561|gene=LOC103468305	FRIM	3458.490	-0.117	0.049

POLARIZATION-SELECTED LINES (n=158)

Transcript	Gene	Average	Fold	P-value

	BLAST	expression	Change	(FDR)
rna18687|gene=tia1	tia1	401.711	0.422	< 0.001
MSTRG.23945.1|gene=MSTRG.23945	CAC1C	1299.068	0.768	< 0.001
rna31577|gene=LOC103477205	TACC2	2488.829	0.178	< 0.001
MSTRG.21916.1|gene=MSTRG.21916	ZEP2	6873.343	0.187	< 0.001
rna9693|gene=abhd14a	abhd14a	1497.664	-0.168	0.001
MSTRG.18769.2|gene=MSTRG.18769	KIF1A	7524.576	0.217	0.001
rna20419|gene=rictor	rictor	558.369	0.353	0.001
rna42366|gene=LOC103457350	MAX	355.081	0.441	0.001
MSTRG.7449.1|gene=MSTRG.7449	AT2B3	4327.516	0.172	0.001
MSTRG.11745.1|gene=MSTRG.11745	P33MX	3551.885	-0.160	0.002
rna15494|gene=LOC103467551	SLMAP	1057.196	-0.220	0.002
rna10910|gene=LOC103464892	SORT1	2240.260	0.317	0.002
rna46476|gene=ppm1e	ppm1e	1440.302	0.315	0.003
rna39999|gene=LOC103482255	PFKAP	882.019	-1.088	0.003
rna39992|gene=LOC103482255	PFKAP	1367.223	0.413	0.003
rna9416|gene=LOC103464216	LRC24	586.747	0.403	0.003
MSTRG.9631.2|gene=MSTRG.9631	ADA11	7045.660	0.126	0.003
rna42290|gene=gphn	gphn	1236.892	0.239	0.006
rna139|gene=pcm1	pcm1	415.579	0.513	0.006
MSTRG.26314.1|gene=MSTRG.26314	LIMS1	248.767	-0.421	0.006
MSTRG.9224.4|gene=MSTRG.9224	GFAP	2397.131	-0.223	0.008
rna21649|gene=atp8a1	atp8a1	223.183	7.673	0.008
rna30694|gene=pgbd5	pgbd5	1308.949	0.466	0.008
MSTRG.14919.1|gene=MSTRG.14919	KLC1	317.188	-0.494	0.009
rna44843|gene=magi2	magi2	333.647	-6.397	0.009
rna4264|gene=s100b	s100b	8575.720	-0.294	0.011
rna8601|gene=LOC103463821	REM2	1266.508	0.215	0.011
MSTRG.5319.1|gene=MSTRG.5319	MTG8R	289.321	0.659	0.011
MSTRG.8385.2|gene=MSTRG.8385	CHDH	613.368	-0.337	0.011
rna35142|gene=LOC103479283	PTPRS	3184.232	0.228	0.011
MSTRG.25460.5|gene=MSTRG.25460	DPYL2	1490.984	-0.159	0.011
rna10217|gene=LOC103464678	S12A5	292.625	0.760	0.011
rna3294|gene=LOC103477739	T22D1	942.977	0.314	0.013
MSTRG.16563.2|gene=MSTRG.16563	KHDR2	596.821	-0.263	0.014
MSTRG.904.5|gene=MSTRG.904	SMCA4	825.667	0.940	0.014
rna7625|gene=LOC103463153	KCC4	813.768	0.306	0.015
rna23887|gene=LOC103472687	RS27	8841.463	-0.132	0.015
rna40585|gene=atp6v1a	atp6v1a	3486.538	0.279	0.015
rna28612|gene=LOC103475568	NBEA	1738.565	0.230	0.015
rna30893|gene=eif4ebp2	eif4ebp2	4206.554	-0.129	0.015

rna22733|gene=LOC103472023	SIA4A	329.212	-0.484	0.016
rna22398|gene=gria2	gria2	1501.733	0.394	0.018
MSTRG.13007.1|gene=MSTRG.13007	DEN1A	523.827	-0.258	0.018
MSTRG.604.1|gene=MSTRG.604	PCD10	853.615	-0.214	0.020
rna20270|gene=LOC103470513	BMP1	704.668	0.216	0.020
MSTRG.8142.2|gene=MSTRG.8142	GRM7	2211.218	0.219	0.020
rna26271|gene=apbb1	apbb1	616.593	-0.529	0.021
rna7160|gene=LOC103463007	SHAN1	1246.491	0.303	0.021
rna38233|gene=jph3	jph3	1481.033	0.154	0.021
rna9994|gene=atp2b2	atp2b2	2968.692	0.160	0.021
MSTRG.7852.1|gene=MSTRG.7852	TM269	642.667	-0.332	0.021
rna41217|gene=u2surp	u2surp	260.347	0.496	0.021
rna16889|gene=smcr8	smcr8	505.442	0.242	0.021
rna20696|gene=LOC103470760	ODO1	3680.969	0.128	0.022
rna6714|gene=madd	madd	581.771	0.781	0.024
rna28842|gene=LOC103475446	PP2BA	1272.204	0.483	0.025
rna18171|gene=crebbp	crebbp	477.352	0.698	0.025
MSTRG.24874.5|gene=MSTRG.24874	LHPL5	1008.636	-0.240	0.025
rna7423|gene=LOC103463224	DDAH1	614.691	-0.294	0.025
rna38076|gene=LOC103481097	MPRIP	1004.516	0.694	0.026
rna15448|gene=bicd2	bicd2	802.184	0.257	0.026
rna21869|gene=mid2	mid2	858.588	-0.264	0.028
rna1133|gene=LOC103463598	S14L1	402.061	0.463	0.028
rna18056|gene=tom1l2	tom1l2	1234.140	-0.201	0.028
rna26413|gene=LOC103474211	ROBO2	957.985	0.457	0.029
rna3034|gene=tmem47	tmem47	663.543	-0.408	0.029
MSTRG.26426.1|gene=MSTRG.26426	AT2B3	509.885	0.223	0.029
rna14675|gene=kif1b	kif1b	6926.073	0.221	0.029
rna28593|gene=pds5b	pds5b	4058.133	0.116	0.029
rna6897|gene=LOC103462737	RGRF1	3742.185	0.223	0.029
rna12565|gene=cacna2d1	cacna2d1	307.267	0.642	0.029
rna25879|gene=LOC103473860	SHC3	917.756	0.542	0.030
MSTRG.20400.1|gene=MSTRG.20400	NETR	2933.854	-1.107	0.030
rna10085|gene=r3hdm2	r3hdm2	882.792	0.293	0.030
rna2232|gene=sh3gl2	sh3gl2	1153.102	0.336	0.030
rna3256|gene=LOC103477319	NA	369.700	-0.551	0.030
rna7150|gene=cttn	cttn	1432.342	0.154	0.030
MSTRG.23582.7|gene=MSTRG.23582	UNC79	2182.014	-0.299	0.031
rna28372|gene=LOC103475354	SEP8A	1479.812	0.221	0.031
rna40511|gene=stau2	stau2	3751.772	0.101	0.031
rna18955|gene=rasgef1b	rasgef1b	314.812	0.469	0.031

rna40847|gene=cnksr2	cnksr2	2782.045	0.236	0.031
rna32682|gene=LOC103477774	NA	5094.213	-0.150	0.031
rna11361|gene=LOC103465345	LRP1	1464.591	0.319	0.031
rna12226|gene=ptprz1	ptprz1	939.568	-0.197	0.032
MSTRG.24892.1|gene=MSTRG.24892	JUN	1541.880	0.159	0.032
rna48421|gene=LOC103461426	PACA	6547.169	0.114	0.032
MSTRG.1426.7|gene=MSTRG.1426	MCF2L	1541.030	0.216	0.032
rna19642|gene=smarcad1	smarcad1	374.477	0.279	0.033
rna45583|gene=LOC103459444	PRIC1	953.356	0.188	0.033
rna9152|gene=ttll7	ttll7	351.793	0.344	0.033
rna9558|gene=rims4	rims4	2057.525	0.123	0.033
rna26824|gene=baz1b	baz1b	250.301	0.419	0.033
rna27920|gene=LOC103475082	CUL5	478.350	0.422	0.034
rna30614|gene=LOC103476702	PKHA1	1179.339	-0.144	0.034
MSTRG.10701.2|gene=MSTRG.10701	DPYL2	293.373	-0.561	0.035
rna22264|gene=LOC103471194	SFXN1	1299.021	-0.161	0.035
rna45277|gene=cand1	cand1	2417.395	0.115	0.035
rna44024|gene=srrm1	srrm1	719.011	0.472	0.035
rna14390|gene=gnb1	gnb1	2102.920	0.133	0.035
rna19155|gene=camsap1	camsap1	366.586	-0.849	0.035
rna11115|gene=LOC103465107	BSN	3020.560	0.361	0.038
rna13371|gene=znf746	znf746	1535.108	0.216	0.038
rna39374|gene=LOC103481898	KGP1	3810.363	0.112	0.038
rna45925|gene=LOC103459684	CDK17	1635.928	0.146	0.038
rna22088|gene=LOC103471305	SSH	1433.568	0.179	0.038
rna9540|gene=LOC103464285	AT2B4	966.284	0.325	0.038
rna7314|gene=LOC103462926	GSLG1	2352.158	0.169	0.039
rna25580|gene=dgcr2	dgcr2	862.995	0.353	0.039
rna19014|gene=ralgds	ralgds	289.546	0.335	0.039
rna46771|gene=rad17	rad17	289.675	-0.260	0.040
rna21459|gene=LOC103471609	ZIC3	848.137	0.211	0.041
rna33285|gene=LOC103478674	T200B	768.169	0.236	0.041
rna29284|gene=klc2	klc2	1027.397	0.157	0.041
rna21130|gene=LOC103471057	BRD8	579.688	0.296	0.041
rna47838|gene=LOC103460885	ATRN1	749.916	0.186	0.042
rna43237|gene=jade1	jade1	486.768	0.251	0.042
rna39189|gene=srcap	srcap	3117.647	0.130	0.043
rna5118|gene=LOC103460319	TM163	2283.059	0.153	0.044
rna40679|gene=arfgef1	arfgef1	1093.914	0.292	0.044
rna36138|gene=LOC108167071	MIB1	579.766	-0.245	0.044
rna33009|gene=gsk3a	gsk3a	390.156	0.245	0.045

rna33558|gene=kmt2b	kmt2b	1100.465	0.334	0.045
rna7409|gene=LOC103463058	LRP8	248.208	0.326	0.045
rna36173|gene=dnajb4	dnajb4	1321.044	0.178	0.045
rna2990|gene=LOC103474271	SCN2A	797.677	0.430	0.045
rna12431|gene=LOC103465918	S6A13	261.539	-0.709	0.045
rna39333|gene=LOC103481878	GRID1	688.931	0.397	0.045
MSTRG.11445.1|gene=MSTRG.11445	TSN7	913.167	-0.359	0.045
rna4111|gene=etv5	etv5	603.824	0.555	0.045
rna14581|gene=LOC103467180	AMPD2	4309.644	0.105	0.045
rna17113|gene=sox8	sox8	1165.511	0.165	0.045
rna6646|gene=LOC103462596	TLN2	1334.136	0.289	0.045
rna1884|gene=acox1	acox1	922.029	-0.142	0.045
MSTRG.11781.2|gene=MSTRG.11781	ATRX	5501.649	0.144	0.045
rna41153|gene=ptprn2	ptprn2	11939.245	0.175	0.045
rna4922|gene=LOC103461631	PTPRN	8049.927	0.206	0.045
rna2054|gene=LOC103470599	TEN3	754.778	-0.460	0.045
rna37531|gene=LOC103480722	PP2BA	3022.883	0.160	0.045
MSTRG.2884.1|gene=MSTRG.2884	CD81	3884.961	-0.100	0.045
rna10431|gene=LOC103464782	HES5	297.115	-0.279	0.045
rna35326|gene=LOC103479394	SCG2	38239.656	0.110	0.045
rna19050|gene=LOC103469848	KCC2B	398.238	0.440	0.046
MSTRG.9859.3|gene=MSTRG.9859	TIA1	386.806	-0.289	0.046
rna2730|gene=app	app	1253.390	0.129	0.046
rna43935|gene=kbtbd11	kbtbd11	3946.524	0.180	0.048
rna22259|gene=gabrb2	gabrb2	2528.544	0.149	0.048
rna28126|gene=hnrnpl	hnrnpl	1361.666	0.189	0.048
rna4001|gene=sumo1	sumo1	1338.464	-0.129	0.049
rna19299|gene=slc20a2	slc20a2	629.265	-0.668	0.049
MSTRG.10579.6|gene=MSTRG.10579	MEF2C	2175.170	-0.305	0.049
rna32394|gene=cspg5	cspg5	7576.875	0.148	0.049
rna12473|gene=syt12	syt12	4226.735	0.156	0.049
rna11522|gene=LOC103465434	CDK18	613.584	0.944	0.049
MSTRG.21665.4|gene=MSTRG.21665	ASAP1	1872.921	-0.200	0.049
MSTRG.8757.1|gene=MSTRG.8757	UBP43	232.268	-0.358	0.049
rna43050|gene=ndufaf4	ndufaf4	394.548	-0.229	0.049
rna28590|gene=fry	fry	756.197	0.748	0.049

Supplementary Table 7. Summary of differentially co-expressed gene pair results from differential coexpression analyses to evaluate the genomic response in midbrain, optic tectum and telencephalon in polarization-selected and control lines of female guppies when swimming with a group of conspecifics versus when swimming alone (two samples per treatment and brain region obtained by pooling brain tissue from 5 individuals each, n = 24 pools).

	OPTIC TECTUM			TELENCEPHALON			MIDBRAIN		
	Control line		Schooling line	Control line		Schooling line	Control line		Schooling line
DC gene pairs	29		65	228		35	67		60
DC unique genes	27		61	161		36	61		55
DC genes in common (between CT and SCH lines)		NA			*ELAV2*			*MKL2*<br>*IGF1R*<br>*TRIM39*<br>*RPL19*<br>*POLR1B*	
Genes that are DC in more than one comparison	*TNIKB ELAVL2 NTMT1 LOC103468557 GRIA2 KCAB1 LBH SURF2*<br>*GBA2 MSTRG.8823 RL19 ARL4A GOLGA5 IGF1R MKL2 POLR1B*<br>*RANBP3 TRI39 FACR1 PARVA PFKFB2 PTGR2*								

Supplementary Table 8. List of differentially coexpressed genes identified by BFDCA by brain region and selection line with number of differentially correlated connections (DC gene pairs) for each gene (two samples per treatment and brain region obtained by pooling brain tissue from 5 individuals each, n = 24 pools). Genes highlighted in green are DC in more than one comparison, and those highlighted in blue are also Diferentially Expressed in GO enrichment tests.

OPTIC TECTUM				TELENCEPHALON				MIDBRAIN			
Control lines		Polarization-selected lines		Control lines		Polarization-selected lines		Control lines		Polarization-selected lines	
ADPRH	4	TLK2	7	mea1	13	slc17a6	4	MAGI1	5	LOC103461370	6
CGAS	4	AP1B1	6	KAT7	7	tenm2	4	csk	4	PLAK	6
ABCA1	3	rtn4	5	ndufs4	7	tm2d1	4	IGF1R	4	mmp16	5
CENPS	3	ZN226	5	apmap	6	clmn	3	PPARA	4	CNTNAP5	4
MSTRG.7285	3	PARVA	4	arhgef4	6	FACR1	3	CL17A	3	CPSF3	4
LOC103468557	3	pfkfb2	4	NIPLB	6	KHDR2	3	F264	3	pou4f1	4
NRIP3	3	SHC3	4	pkn1	6	LRC24	3	hmga2	3	abracl	3
pcdh19	3	tom1l2	4	rpl17	6	PTPRS	3	LOC103468544	3	CCDC127	3
STIMA	3	ASIC1	3	TRIM69	6	SUIS_HUMAN	2	LOC103468557	3	DNAI2	3
SURF2	3	CCDC51	3	ARIP4	5	grm1	2	MILK1	3	HES6	3
BC11B	2	CDH6	3	cndp2	5	LBH	2	polr1b	3	LOC103461363	3
DLGAP2	2	ERLN1	3	ldhb	5	MACF1	2	RASSF5	3	NUP205	3
ENAH	2	GRID2	3	LRRT4	5	MSTRG.5809	2	rbm33	3	otp	3
GBA2	2	KCC2B	3	rps8	5	mthfs	2	rbm45	3	pdgfrb	3
KCNH7	2	KLHL17	3	smarca4	5	nrbp2	2	S4A7	3	ABRAL	2
mapk6	2	mphosph8	3	tbc1d13	5	PNN	2	ABCD1	2	ARI1B	2
rbms1	2	ntmt1	3	AT8B2	4	RAB3A	2	dctn1	2	ARL4A	2
slc29a2	2	r3hdm2	3	CYTB	4	rheb	2	dnal1	2	ARL6	2
tmbim6	2	TSPAN8	3	dvl1	4	rpl7	2	DNPH1	2	CALB2	2
ADHX	1	cdk14	2	gatad2b	4	RXRBA	2	DOCK9	2	clg20hxorf23	2
ARL4A	1	CROL2	2	htr7	4	TLK1B	2	eaf1	2	CORO2A	2
clns1a	1	EAF1	2	ier2	4	TM205	2	GRIA2	2	ctps1	2
SCD5	1	FOXP2	2	itgb5	4	ABLIM2	1	grip1	2	gapdhs	2
sephs1	1	HAPLN1	2	jarid2	4	CSK21	1	HMCS1	2	golga5	2
slc39a7	1	HDGFL3	2	NUCB2	4	elavl2	1	IPKA	2	GTDC1	2
tnikb	1	HOME3	2	PCDHGA2	4	LYSC	1	itpkb	2	IGF1R	2
ZN235	1	LOC103459553	2	rabgap1	4	MAPK9	1	KCAB1	2	KCNJ9	2
		lrrtm4l1	2	rpl14	4	MSTRG.8823	1	LBH	2	LOC103460906	2
		NPAS2	2	SATT	4	ntmt1	1	LOC103476863	2	mkl2	2
		NRP1A	2	scai	4	PP2BA	1	LORF2	2	nrg2	2
		NSG2	2	SCN5A	4	ppp1r13l	1	LRRF2	2	NTM1B	2
		PHF1	2	tbl1xr1	4	PTGR2	1	map6d1	2	polr1b	2
		ptprd	2	tmem145	4	thumpd2	1	mkl2	2	ranbp3	2
		st3gal4	2	tnrc6b	4	TRAPPC1	1	nfe2l1	2	RL19	2
		ube2v2	2	ZN865	4	TSP1L	1	nlgn2	2	rpl37a	2
		ALDH18A1	1	aars2	3	zmynd12	1	NPRL3	2	SEPTIN12	2
		APBA1	1	ache	3			pfkp	2	SFMBT1	2
		cfap99	1	AGRN	3			PKIA	2	sin3a	2
		CHD8	1	AMT	3			PRC1	2	TLE4	2
		cpeb4	1	ANK2	3			prickle2	2	TRI39	2
		EIF4G3	1	ASAP2	3			rexo1	2	abcg4	1
		GLSK	1	ATX1	3			rpl15	2	cdc42bpb	1
		GLUL	1	bdnf	3			SURF2	2	COR1A	1
		GNG7	1	bicd2	3			thap5	2	FACR1	1
		heatr5b	1	cacna1a	3			tnikb	2	gdpd2	1
		ITPR1	1	cdipt	3			TRI39	2	IL1A	1
		kiaa2026	1	COX18	3			TSPAN7	2	MRPL55	1
		LOC103464370	1	cpsf6	3			TUFT1	2	MSTRG.24943	1
		LOC103477566	1	csgalnact2	3			ZFP2	2	ncoa4	1
		NCAM2	1	DDX42	3			atp2b2	1	orc4	1
		NR1AA	1	dnmbp	3			CNGB1	1	PARVA	1
		nt5e	1	dtnb	3			DCNL1	1	pfkfb2	1
		orc5	1	fau	3			elavl4	1	pla1a	1
		ptprm	1	FLRT1	3			GBA2	1	PTGR2	1
		ranbp3	1	GRIA2	3			grk4	1	RHG22	1
		rpl8	1	HACE1	3			MSTRG.8823	1		
		RTase	1	HDAC4	3			MYO1E	1		

SRSF5	1	HS6ST2	3	*ran*	1
TSC22D2	1	hypk	3	*RL19*	1
vps45	1	KCD15	3	*rundc3a*	1
XPC	1	MAGI2	3	*SCHIP1*	1
		nell1	3	*shroom2*	1
		NTRK2	3	*SYPH*	1
		NYNRIN	3	*tanc2*	1
		P85B	3	*znf512*	1
		PMM2	3		
		psmc3	3		
		RAC3	3		
		RADIL	3		
		RL19	3		
		rpl10	3		
		rpl11	3		
		rpl30	3		
		rpl7a	3		
		rps3a	3		
		SCAM5	3		
		slc1a1	3		
		SPAG9	3		
		sptbn4	3		
		SYGP1	3		
		TACC1	3		
		tmem19	3		
		TRPC4AP	3		
		UBA3	3		
		USP9X	3		
		wdr18	3		
		zcchc6	3		
		zyg11b	3		
		AN13C	2		
		AN32E	2		
		atg3	2		
		B3GL2	2		
		CHD5	2		
		csmd2	2		
		EF1A1	2		
		EVL	2		
		GNAQ	2		
		golga5	2		
		GRM5	2		
		KCNH8	2		
		kdm4a	2		
		LAP2	2		
		LOC103460099	2		
		LOC103467299	2		
		lrrc75a	2		
		LRRT2	2		
		marcksl1	2		
		ncln	2		
		NLGN1	2		
		NOVA1	2		
		NTL9	2		
		PAK1IP1	2		
		pfdn6	2		
		PHLPP2	2		
		ppargc1b	2		
		PPP2R5C	2		
		prkca	2		
		RL12	2		
		rpl31	2		
		rplp0	2		
		S4A11	2		
		SCN8A	2		
		SI1L1	2		
		slc1a2	2		
		slc6a11	2		
		spen	2		
		SPTBN4	2		

	TESK2	2	
	TMTC3	2	
	TRPM6	2	
	uba52	2	
	UBC2	2	
	UNC13A	2	
	XPO1	2	
	ZN665	2	
	ZNF521	2	
	znf839	2	
	AHNK	1	
	BMTI6	1	
	BSN	1	
	ctsa	1	
	elavl2	1	
	GP155	1	
	GPR37	1	
	hspd1	1	
	KCAB1	1	
	LOC103460110	1	
	LOC103460981	1	
	lrig1	1	
	lrrc8a	1	
	MYT1	1	
	ndufa11	1	
	PLXA2	1	
	ptprb	1	
	radil	1	
	SHSA6	1	
	snrk	1	
	snrpa	1	
	SNTG1	1	
	tnikb	1	
	TRI46	1	

Supplementary Table 9. Summary of genes found to be differentially co-expressed and differentially expressed in differential coexpression analyses and GO enrichment analyses in tests respectively, performed to evaluate the genomic response in midbrain, optic tectum and telencephalon in polarization-selected and control lines of female guppies when swimming with a group of conspecifics versus when swimming alone (two samples per treatment and brain region obtained by pooling brain tissue from 5 individuals each, n = 24 pools).

COMMON DE & DC GENES					
OPTIC TECTUM		TELENCEPHALON		MIDBRAIN	
CONTROL	POLARIZATION	CONTROL	POLARIZATION	CONTROL	POLARIZATION
3	0	2	4	3	0
SEPHS1<br>*ZN235*<br>*ARL4A*		*NCLN*<br>*PTPRB*	*LRRC24*<br>*PTPRS*<br>*KHDR2*<br>*PP2BA*	*IGF1R*<br>*ITPKB*<br>*TANC2*	

Supplementary Table 10. KEGG pathways of interest associated in BFDCA differentially coexpressed gene pairs obtained in differential coexpression analyses perfomed to evaluate the genomic response in midbrain, optic tectum and telencephalon in polarization-selected and control lines of female guppies when swimming with a group of conspecifics versus when swimming alone (two samples per treatment and brain region obtained by pooling brain tissue from 5 individuals each, n = 24 pools). Only showing pathways and select genes relevant to neural processes associated with behaviour that presented a FDR corrected p-value <0.05

**OPTIC TECTUM**	
**Polarization-selection lines**	
Pathways of interest (enriched)	Genes of interest
Glutamatergic synapse	GLUL,GNG7,ITPR1
Long-term depression	GRID2,ITPR1
Dopaminergic synapse	GNG7,ITPR1
GABAergic synapse	GLUL,GNG7
Cholinergic synapse	GNG7,ITPR1
Serotonergic synapse	GNG7,ITPR1
**Control lines**	
Signal Transduction of S1P Receptor	MAPK6
**TELENCEPHALON**	
**Polarization-selection lines**	
Pathways of interest (enriched)	Genes of interest
FoxO signaling pathway	GRM1,MAPK9
Phospholipase D signaling pathway	GRM1,RHEB
Synaptic vesicle cycle	SLC17A6,RAB3A
Glutamatergic synapse	SLC17A6,GRM1
JNK (c-Jun kinases) phosphorylation and activation	MAPK9
mediated by activated human TAK1	RAB3A
GABA synthesis, release, reuptake and degradation	RAB3A
Glutamate Neurotransmitter Release Cycle	
**Control lines**	
Glutamatergic synapse	CACNA1A,GRIA2,SLC1A1,GNAQ,GRM5,PRKCA,SLC1A2
Synaptic vesicle cycle	CACNA1A,SLC1A1,SLC1A2,SLC6A11,UNC13A
PI3K-Akt signaling pathway	PKN1,ITGB5,BDNF,MAGI2,NTRK2,PHLPP2,PPP2R5C,PRKCA
Long-term depression	CACNA1A,GRIA2,GNAQ,PRKCA
Dopaminergic synapse	CACNA1A,GRIA2,GNAQ,PPP2R5C,PRKCA
Long-term potentiation	GRIA2,GNAQ,GRM5,PRKCA
Cholinergic synapse	ACHE,CACNA1A,GNAQ,PRKCA
Ras signaling pathway	HTR7,BDNF,NTRK2,RAC3,PRKCA
**MIDBRAIN**	
**Polarization-selection lines**	
EGFR tyrosine kinase inhibitor resistance	PDGFRB,IGF1R,NRG2
Ras signaling pathway	PDGFRB,IGF1R,PLA1A
MAPK signaling pathway	PDGFRB,IGF1R,IL1A
AMPK signaling pathway	IGF1R,PFKFB2
**Control lines**	
cAMP signaling pathway	PPARA,GRIA2,ATP2B2,CNGB1

Rap1 signaling pathway	MAGI1,IGF1R,RASSF5
Long-term depression	IGF1R,GRIA2
AMPK signaling pathway	IGF1R,PFKP
cGMP-PKG signaling pathway	ATP2B2,CNGB1
Glutamate binding, activation of AMPA receptors and synaptic plasticity	GRIA2,GRIP1,TSPAN7

Supplementary Table 11. Gene Ontology (GO) terms of interest associated with BFDCA DC gene pairs

OPTIC TECTUM	
**Polarization-selected lines**	
GO terms of interest (enriched)	Genes of interest
response to stimulus	TLK2,RTN4,PARVA,PFKFB2,SHC3,TOM1L2,ASIC1,CDH6,GRID2,TSPAN8,CDK14,FOXP2,HDGFL3,NPAS2,NSG2,PHF1,PTPRD,ST3GAL4,UBE2V2,CHD8,CPEB4,GLUL,GNG7,ITPR1,NT5E,PTPRM,SRSF5,TSC22D2,VPS45,XPC
signaling	TLK2,RTN4,PFKFB2,SHC3,TOM1L2,ASIC1,CDH6,GRID2,CDK14,HDGFL3,NSG2,PTPRD,APBA1,CHD8,CPEB4,GLUL,GNG7,ITPR1,NT5E,PTPRM,XPC
response to stress	TLK2,RTN4,ASIC1,TSPAN8,NPAS2,PHF1,ST3GAL4,UBE2V2,CPEB4,GLUL,ITPR1,NT5E,TSC22D2,VPS45,XPC
signal transduction	TLK2,RTN4,SHC3,TOM1L2,ASIC1,CDH6,GRID2,CDK14,HDGFL3,NSG2,PTPRD,CHD8,CPEB4,GNG7,ITPR1,NT5E,PTPRM,XPC
cell-cell signaling	PFKFB2,ASIC1,GRID2,CDK14,PTPRD,APBA1,CHD8,GLUL,ITPR1
response to external stimulus	PARVA,SHC3,GRID2,TSPAN8,ST3GAL4,CHD8,CPEB4,GLUL,NT5E,PTPRM,XPC
synapse	RTN4,ASIC1,GRID2,KLHL17,HAPLN1,PTPRD,APBA1,CPEB4,GLUL,ITPR1,RPL8,VPS45
postsynaptic density	RTN4,GRID2,KLHL17,CPEB4,ITPR1,RPL8
asymmetric synapse	RTN4,GRID2,KLHL17,CPEB4,ITPR1,RPL8
postsynaptic specialization	RTN4,GRID2,KLHL17,CPEB4,ITPR1,RPL8
postsynapse	RTN4,GRID2,KLHL17,APBA1,CPEB4,ITPR1,RPL8
**Control lines**	
response to stimulus	CGAS,ABCA1,CENPS,STIMATE,BCL11B,DLGAP2,ENAH,MAPK6,TMBIM6,ADH5
positive regulation of cGMP-mediated signaling	CGAS
neurogenesis	BCL11B,ENAH,GBA2,MAPK6
response to L-glutamate	TMBIM6
response to external stimulus	CGAS,ABCA1,BCL11B,ENAH,ADH5
regulation of signal transduction	CGAS,ABCA1,STIMATE,DLGAP2,TMBIM6
neurotransmitter reuptake	SLC29A2
**TELENCEPHALON**	
**Polarization-selected lines**	

L-glutamate transmembrane transporter activity	SLC17A6, GRM1
neurotransmitter transmembrane transporter	SLC17A6
activity	GRM1
glutamate receptor activity	GRM1
postsynaptic neurotransmitter receptor activity	SLC17A6, MACF1, RAB3A,
L-glutamate transmembrane transport	RAB3A, MACF1
evoked neurotransmitter secretion	MAPK9
protein localization to tricellular tight junction	MAPK9
JUN phosphorylation	PTPRS
regulation of cell junction assembly	SLC17A6
neurotransmitter loading into synaptic vesicle	GRM1
adenylate cyclase-inhibiting G protein-coupled	
glutamate receptor signaling pathway	SLC17A6
neurotransmitter transport	GRM1, MACF1
L-glutamate import across plasma membrane	GRM1
G protein-coupled glutamate receptor signaling	MAPK9
pathway	PTPRS, TENM2, PTPRS, GRM1, MACF1, PNN,
protein localization to cell-cell junction	RAB3A, RHEB, RPL7, PPP1R13L
cell junction assembly	MTHFS
	GRM1
glutamate metabolic process	
glutamate receptor signaling pathway	
clathrin-sculpted glutamate transport vesicle	RAB3A
membrane	RAB3A
clathrin-sculpted glutamate transport vesicle	PNN
exon-exon junction complex	PTPRS
glutamatergic synapse	
**Control lines**	
high-affinity glutamate transmembrane transporter	SLC1A1,SLC1A2
activity	SLC1A1,SLC1A2
glutamate:sodium symporter activity	KAT7,PKN1,SMARCA4,DVL1,GATAD2B,IER2,JARI
aromatic compound biosynthetic process	D2,SCAI,TBL1XR1,TNRC6B,AGRN,HDAC4,PMM2,P
	SMC3,RPL10,TACC1,UBA3,USP9X,CHD5,KDM4A,P
	PARGC1B,SPEN,UBA52,XPO1,ZNF521,ELAVL2,GP
	R37,HSPD1,MYT1
synapse organization	DVL1,ACHE,AGRN,BDNF,NTRK2,SLC1A1,GRM5,N
glutamate receptor signaling pathway	LGN1,UNC13A,BSN
neurotransmitter transport	GRIA2,SLC1A1,GNAQ,GRM5
	DVL1,SLC1A1,NLGN1,SLC1A2,SLC6A11,UNC13A
**MIDBRAIN**	
**Polarization-selected lines**	

protein tyrosine kinase activity	PDGFRB,IGF1R
cellular aromatic compound metabolic process	CPSF3,POU4F1,HES6,NUP205,OTP,PDGFRB,CTPS1,
	GAPDHS,MRTFB,POLR1B,RPL19,RPL37A,SFMBT1,
aromatic compound biosynthetic process	SIN3A,TLE4,IL1A,NCOA4,ORC4,PFKFB2
	CPSF3,POU4F1,HES6,OTP,PDGFRB,CTPS1,MRTFB,
inactivation of MAPKK activity	POLR1B,SFMBT1,SIN3A,TLE4,IL1A,NCOA4
signal transduction	IGF1R
	POU4F1,PDGFRB,ARL6,CORO2A,IGF1R,NRG2,TLE
equilibrioception	4,TRIM39,CDC42BPB,IL1A,NCOA4
regulation of signal transduction	POU4F1
suckling behavior	POU4F1,PDGFRB,IGF1R,NRG2,TLE4,TRIM39,IL1A
	POU4F1
**Control lines**	
signaling receptor activity	CSK,IGF1R,PPARA,GRIA2,NLGN2
signal transduction	MAGI1,CSK,IGF1R,PPARA,HMGA2,RASSF5,DCTN1
behavior	,DOCK9,GRIA2,GRIP1,ITPKB,LBH,NLGN2,NPRL3,P
glutamatergic synapse	RICKLE2,TUFT1,CNGB1,GRK4,MYO1E,RUNDC3A,
AMPA glutamate receptor complex	SCHIP1
	PPARA,DCTN1,NLGN2,ELAVL4
	GRIP1,ATP2B2,ELAVL4
	GRIA2

Supplementary References

1 - Mi, H., Muruganujan, A., Casagrande, J. T. & Thomas, P. D. Large-scale gene function analysis with the PANTHER classification system. *Nature Protocols 2013 8:8* **8**, 1551–1566 (2013).

2 - Supek, F., Bošnjak, M., Škunca, N. & Šmuc, T. REVIGO summarizes and visualizes long lists of gene ontology terms. *PLoS One* **6**, (2011).
