# Supplementary material for: Functional convergence of genomic and transcriptomic architecture underlies schooling behaviour in a live-bearing fish
Source: Nat Ecol Evol. 2023 Nov 20;8(1):98–110. doi: 10.1038/s41559-023-02249-9 (PMC10781616; doi:10.1038/s41559-023-02249-9)
Supplement: Supplementary file 2 — Reporting Summary [file 41559_2023_2249_MOESM2_ESM.pdf]

nature portfolio

Corresponding author(s): Alberto Corral Lopez

Last updated by author(s): Sep 25, 2023

Reporting Summary

Nature Portfolio wishes to improve the reproducibility of the work that we publish. This form provides structure for consistency and transparency in reporting. For further information on Nature Portfolio policies, see our Editorial Policies and the Editorial Policy Checklist.

Statistics

For all statistical analyses, confirm that the following items are present in the figure legend, table legend, main text, or Methods section.

n/a	Confirmed	
☐	☒	The exact sample size (*n*) for each experimental group/condition, given as a discrete number and unit of measurement
☐	☒	A statement on whether measurements were taken from distinct samples or whether the same sample was measured repeatedly
☐	☒	The statistical test(s) used AND whether they are one- or two-sided<br>*Only common tests should be described solely by name; describe more complex techniques in the Methods section.*
☐	☒	A description of all covariates tested
☐	☒	A description of any assumptions or corrections, such as tests of normality and adjustment for multiple comparisons
☐	☒	A full description of the statistical parameters including central tendency (e.g. means) or other basic estimates (e.g. regression coefficient) AND variation (e.g. standard deviation) or associated estimates of uncertainty (e.g. confidence intervals)
☐	☒	For null hypothesis testing, the test statistic (e.g. *F*, *t*, *r*) with confidence intervals, effect sizes, degrees of freedom and *P* value noted<br>*Give P values as exact values whenever suitable.*
☐	☒	For Bayesian analysis, information on the choice of priors and Markov chain Monte Carlo settings
☐	☒	For hierarchical and complex designs, identification of the appropriate level for tests and full reporting of outcomes
☐	☒	Estimates of effect sizes (e.g. Cohen's *d*, Pearson's *r*), indicating how they were calculated

Our web collection on statistics for biologists contains articles on many of the points above.

Software and code

Policy information about availability of computer code

Data collection	Collection motion : idTracker (v2.1)
Data analysis	R (v4.1.3), RStudio (v2022.07.0), Matlab Compiler Runtime 8.3, Matlab (v2020a)<br>Sequence quality control: FastQC (v.0.11.4.553); Trimmomamtic (v0.35)<br>Genome-assembly: HiSat (v2.05); StringTie (v1.32)<br>Pool-Seq: Samtools (v.1.6.0); Popoolation2 (v1.1013)<br>Differential expression and Coexpression: DeSeq2 & BFCSA R packages<br>Functional analyses: PANTHER (v18.0); REVIGO (v1.8.1); gProfiler R package

For manuscripts utilizing custom algorithms or software that are central to the research but not yet described in published literature, software must be made available to editors and reviewers. We strongly encourage code deposition in a community repository (e.g. GitHub). See the Nature Portfolio guidelines for submitting code & software for further information.

Data

Policy information about availability of data

All manuscripts must include a data availability statement. This statement should provide the following information, where applicable:

- Accession codes, unique identifiers, or web links for publicly available datasets
- A description of any restrictions on data availability
- For clinical datasets or third party data, please ensure that the statement adheres to our policy

Data needed to evaluate the conclusions in the paper are deposited in figshare database under accession code 10.6084/m9.figshare.23805702. Genomic and transcriptomic data are deposited at NCBI database under accession codes PRJNA994132 and PRJNA504011. Source data are provided with this paper. Video recordings related to this paper may be requested from the authors.

Research involving human participants, their data, or biological material

Policy information about studies with human participants or human data. See also policy information about sex, gender (identity/presentation), and sexual orientation and race, ethnicity and racism.

Reporting on sex and gender	N/A
Reporting on race, ethnicity, or other socially relevant groupings	N/A
Population characteristics	N/A
Recruitment	N/A
Ethics oversight	N/A

Note that full information on the approval of the study protocol must also be provided in the manuscript.

Field-specific reporting

Please select the one below that is the best fit for your research. If you are not sure, read the appropriate sections before making your selection.

☐ Life sciences ☐ Behavioural & social sciences ☒ Ecological, evolutionary & environmental sciences

For a reference copy of the document with all sections, see nature.com/documents/nr-reporting-summary-flat.pdf

Ecological, evolutionary & environmental sciences study design

All studies must disclose on these points even when the disclosure is negative.

Study description	We phenotyped male and female guppies following artificial selection for higher polarization to estimate the effect on social interactions and the heritability of sociability.<br><br>We sequenced DNA pools (Pool-seq) to identify genome-wide differences in allele frequencies between polarization-selected female guppies that presented high sociability and female guppies that presented low sociability.<br><br>We use RNA sequencing to determine differences in gene expression in multiple brain regions of polarization-slelected and control females in response to two different social contexts (swimming alone or schooling in a group with seven unfamiliar females).
Research sample	Heritability: 1487 guppies across 195 families, including the mother, father, three female and three male offspring from replicated experimental selection lines (three polarization-selected and three control lines)<br><br>Pool-seq: for each replicated selection line, pooled samples from seven females representing top and bottom 20% sociability scores (6 total pooled samples).<br><br>Transcriptomics: pooled samples of brain tissue (optic tectum, midbrain and telencephalon) of ten individuals into two non-overlapping pools of five for each replicated selection line for each treatment (alone or swimming in a group with conspecifics).
Sampling strategy	Heritability: Using offspring of the F3 generation of artificial selection we bred 35 families for each of replicated selection line (three polarization-selected and thre control). We used male and females of same age (9 months old approximately) and paired them in 3L tanks to generate a parental generation. We collected offspring for the two first clutches of these pairs and phenotyped three females and three males at 5-6 moths old.

Genomics: Only samples from parental generation from heritability experiments were kept for subsequent genomic analyses. Based on sociability measurements, we used top and bottom 20% samples from each replicate (7 samples per pool - total 6 pools).

Transcriptomics: This strategy was applied to reduce noise in transcript expression data during sample normalization procedures (because of potential outliers in behavioral experiments) and to maintain each replicate as a comparable unit.

Data collection

Heritability: A.C-L. performed experiments to record social interactions of guppies. Groups of 8 sexually mature female or male guppies were evaluated in an experimental arena using an open field test. An even number of up to 16 groups of fish were tested per day, balancing the number of polarization-selected and control groups per day. All trials were recorded and we tracked positional data of focal fish and mean values for the other 7 individuals in the test. Collective motion characteristics were extracted from tracking data.

Genomics: A.C-L. and M.C-C. extracted DNA from female samples kept in ethanol. We achieved a minimum of 3g genomic DNA per pool using Nextera DNAFlex library preparation kit (Illumina) following manufacturer protocol. The final library containing six pooled samples was sequenced at SciLife Lab, Uppsala.

Transcriptomics: A.K. and S.D.B. performed experiments and dissections for tissue collection. Fish were placed individually or in groups of 8 unfamiliar adult control and polarization-selected females in white 55cm arenas. After 30 minutes, females were euthanized by transfer to ice water and tissues of interest dissected out and kept in RNA later. N.B. extracted RNA by pooling tissue of interest, homogenizing and following Qiagen RNeasy's protocol. libraries for each sample were prepared and sequenced by the Welcome Trust for Human Genetics (Oxford University, UK).

Timing and spatial scale

Heritability: Data collection of parental generation was performed between March-April 2019 (8-16 trials a day - total 390 trials). We stopped data collection for 3-4 months and proceed when individuals of offspring generation sexually matured. Data collection of offspring generation was performed between September-December 2019 (8-16 trials a day - 1100 individuals)

Transcriptomics: measurements and experiments were performed during Autumn 2019 (8 individuals per day during a three-weeks collection period).

All experiments were performed in lab facilities at Department of Zoology, Stockholm University (Sweden).

Data exclusions

Families that did not produce three female and three male offspring were disregarded from behavioral testing. We obtained data for a minimum of 30 families for each of the six selection lines (three polarization-selected, three control).

Following automated tracking, all trials with less than 70% complete tracks (n= 8) were disregarded for further analyses.

Reproducibility

All experiments performed to polarization-selected and control lines relied on protocols successfully applied in previous investigations in this species and setup (e.g. Kotrschal, Szorkovsky et al. Sci Adv 2020). Sampling design of transcriptomic analyses was based on a successful protocol we previously used in the study of neurogenomics of mate preference in guppies (Bloch et al. Nat Eco Evo 2018). There were no attempts to repeat the experiments. Trials with incomplete data were excluded as stated above.

Randomization

Fish used in F0 of our artificial selection procedure were a mix of adult fish of various ages from the lab breeding stock. In subsequent generations we used relatively uniform young adults to minimize time between generations. This design ensured that within every replicate, polarization and control fish were of same age. Fish used for experiments in this study are offspring of similar age from selection and control lines of F3 generation.

An even number of up to 16 groups were tested per day in our experimental setups, balancing the number of polarization-selected and control groups per day

Blinding

Data acquisition was based on extraction from positional data using an automated protocol.

Did the study involve field work? ☐ Yes ☒ No

Reporting for specific materials, systems and methods

We require information from authors about some types of materials, experimental systems and methods used in many studies. Here, indicate whether each material, system or method listed is relevant to your study. If you are not sure if a list item applies to your research, read the appropriate section before selecting a response.

Materials & experimental systems

n/a	Involved in the study
☒	☐ Antibodies
☒	☐ Eukaryotic cell lines
☒	☐ Palaeontology and archaeology
☐	☒ Animals and other organisms
☒	☐ Clinical data
☒	☐ Dual use research of concern
☒	☐ Plants

Methods

n/a	Involved in the study
☒	☐ ChIP-seq
☒	☐ Flow cytometry
☒	☐ MRI-based neuroimaging

Animals and other research organisms

Policy information about studies involving animals; ARRIVE guidelines recommended for reporting animal research, and Sex and Gender in Research

Laboratory animals	Guppies used in our experiment are laboratory-raised descendants of Trinidad guppies sampled from the high predation populations of the Quare River (Trinidad). Generation 0 of selection was a mix of adult fishes of various ages from the laboratory breeding stock, while for the next generations we used relatively uniform young adults (approximately 4-5 months-old).
Wild animals	The study did not involve wild animals
Reporting on sex	We used a breeding design involving male and female guppies to quantify the effect of experimental evolution of higher polarization. We report sex differences in behavioral measurements, as well as across sex correlations in heritability estimates.<br><br>As, we used female guppies as the target of directional selection for higher coordination, genomic and transcriptomic studies presented here focus only on female guppies.
Field-collected samples	The study did not involve samples collected from the field
Ethics oversight	All experiments were performed in accordance with ethical applications approved by the Stockholm Ethical Board (Dnr:C50/12, N173/13, and 223/15). These applications are consistent with the Institutional Animal Care and Use Committee guidelines.

Note that full information on the approval of the study protocol must also be provided in the manuscript.
